# ENDORSE: a prognostic model for endocrine therapy in estrogen‐receptor‐positive breast cancers

**DOI:** 10.15252/msb.202110558

**Published:** 2022-06-07

**Authors:** Aritro Nath, Adam L Cohen, Andrea H Bild

**Affiliations:** ^1^ Department of Medical Oncology and Therapeutics City of Hope Comprehensive Cancer Center Monrovia CA USA; ^2^ Neuro Oncology Program Inova Schar Cancer Institute Fairfax VA USA

**Keywords:** biomarker, cox proportional hazards model, endocrine resistance, lasso, survival analysis, Biomarkers, Cancer, Computational Biology

## Abstract

Advanced and metastatic estrogen receptor‐positive (ER^+^) breast cancers are often endocrine resistant. However, endocrine therapy remains the primary treatment for all advanced ER^+^ breast cancers. Treatment options that may benefit resistant cancers, such as add‐on drugs that target resistance pathways or switching to chemotherapy, are only available after progression on endocrine therapy. Here we developed an endocrine therapy prognostic model for early and advanced ER^+^ breast cancers. The endocrine resistance (ENDORSE) model is composed of two components, each based on the empirical cumulative distribution function of ranked expression of gene signatures. These signatures include a feature set associated with long‐term survival outcomes on endocrine therapy selected using lasso‐regularized Cox regression and a pathway‐based curated set of genes expressed in response to estrogen. We extensively validated ENDORSE in multiple ER^+^ clinical trial datasets and demonstrated superior and consistent performance of the model over clinical covariates, proliferation markers, and multiple published signatures. Finally, genomic and pathway analyses in patient data revealed possible mechanisms that may help develop rational stratification strategies for endocrine‐resistant ER^+^ breast cancer patients.

## Graphical abstract

ENDORSE is an expression signature‐based prognostic model that can predict response to endocrine therapy in ER^+^ breast cancers. It outperforms other prognostic signatures and clinical factors in early and advanced metastatic ER^+^ breast cancers.

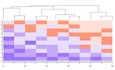

## Introduction

Breast cancer is the most common form of cancer globally, with more than two million cases diagnosed in 2020 (Sung *et al*, 2021). Pathogenesis and classification of breast cancer is based on the presence or absence of estrogen receptor alpha (ER), progesterone receptor (PR), and human growth factor‐neu receptor (HER2). These subtypes guide the selection of systemic therapy for breast cancer patients. More than 70% of breast cancers express ER and are negative for HER2 (ER^+^/HER2^−^) (Harvey *et al*, 1999; Kohler *et al*, 2015). The primary systemic therapy for ER^+^/HER2^−^ breast cancer is endocrine therapy, which counters the growth of tumors by targeting their dependency on estrogen signaling (Waks & Winer, 2019). These therapies include selective estrogen receptor modulators (SERMs) such as tamoxifen and selective estrogen receptor degraders (SERDs) such as fulvestrant that directly prevent ER activation, or aromatase inhibitors like exemestane and anastrozole that reduce circulating levels of estrogen in the body (Smith & Dowsett, 2003; McDonnell & Wardell, 2010). Endocrine therapy substantially reduces the risk of recurrence within 5 years, although chemotherapy may be recommended for some patients with a high risk of recurrence. While clinicopathological features are not reliable predictors of recurrence risk, gene expression‐based genomic tests that predict the risk of recurrence can aid in deciding whether the benefit of adding chemotherapy outweighs its side effects in certain patients (Cardoso *et al*, 2016; Sparano *et al*, 2018). These biomarkers have been validated and recommended for clinical use only in early stage, node‐negative cancers based on guidelines from the American Society of Clinical Oncology and European Group on Tumor Markers (Duffy *et al*, 2017; Krop *et al*, 2017).

Locally advanced and metastatic ER^+^ breast cancers often develop resistance to endocrine therapy with significantly higher rates of recurrence and death compared to early‐stage disease. Despite these challenges, single‐agent endocrine therapy or in combination with CDK4/6 inhibitors remains the primary systemic therapy recommended for locally advanced and metastatic breast cancers (McAndrew & Finn, 2020). Patients may benefit from the addition of a targeted inhibitor against the MTOR or PI3K pathways (Baselga *et al*, 2012; André *et al*, 2019) or switching to chemotherapy (McAndrew & Finn, 2020). However, these treatment options are recommended for consideration only upon progression on endocrine therapy, according to the American Society for Clinical Oncology (Rugo *et al*, 2016), National Comprehensive Cancer Network (Gradishar *et al*, 2017, 2020) and European Society for Medical Oncology (Cardoso *et al*, 2020) clinical practice guidelines. Therefore, the ability to predict the potential benefit from first‐line endocrine therapy may be crucial for locally advanced and metastatic ER^+^ breast cancers that may benefit from continued endocrine therapy, a combination treatment or chemotherapy as the primary treatment strategy.

Unlike early stage, node‐negative disease, genomic tests for endocrine therapy response are not available for advanced and metastatic ER^+^ breast cancers. To address this limitation, a few studies have assessed the genomic signature of endocrine response in ER^+^ metastatic breast cancers (ER^+^ MBC) (Jeselsohn *et al*, 2016; Sinn *et al*, 2019). The TransCONFIRM trial evaluated the transcriptomes of 112 ER^+^/HER2^−^ MBCs and identified a set of 37 genes that were associated with progression‐free survival (PFS) of patients receiving fulvestrant (Jeselsohn *et al*, 2016). Another study analyzed the transcriptomes of 140 ER^+^/HER2^−^ MBC on endocrine therapy to develop SET ER/PR, an 18‐gene predictive score for endocrine therapy sensitivity (Sinn *et al*, 2019). While both the TransCONFIRM and SET ER/PR biomarkers predicted endocrine response in their respective training datasets, neither study performed systematic validation of their predictive signatures to demonstrate the reproducibility and accuracy in independent clinical datasets. This issue highlights a critical flaw in biomarker development pipelines and is one important reason why genomic biomarkers are infrequently translated into clinical practice (Boutros, 2015). Another pervasive issue hindering clinical translation arises from the reliance on a large number of predictive features in complex models that are difficult to interpret and often perform poorly in independent validation due to overfitting (Taylor *et al*, 2008; Witten & Tibshirani, 2010).

Here we developed ENDORSE: a low‐dimensional gene expression‐based prognostic model for endocrine therapy, and systemically tested its performance and predictive ability in multiple clinical trial datasets. ENDORSE was developed using the tumor transcriptomes and overall survival (OS) of more than 800 ER^+^ breast cancers on endocrine therapy (Curtis *et al*, 2012; Pereira *et al*, 2016). We evaluated the performance of ENDORSE compared to clinical covariates, proliferation markers, and other published signatures. We validated the ENDORSE model in multiple independent clinical trial datasets, including the TransCONFIRM and SET ER/PR trials for endocrine therapy in metastatic ER^+^ breast cancer. Our results show that ENDORSE consistently identified high‐risk patients and outperformed all other prognostic models in ER^+^ breast cancers.

## Results

### Developing a prognostic model for endocrine therapy

**Figure 1:**
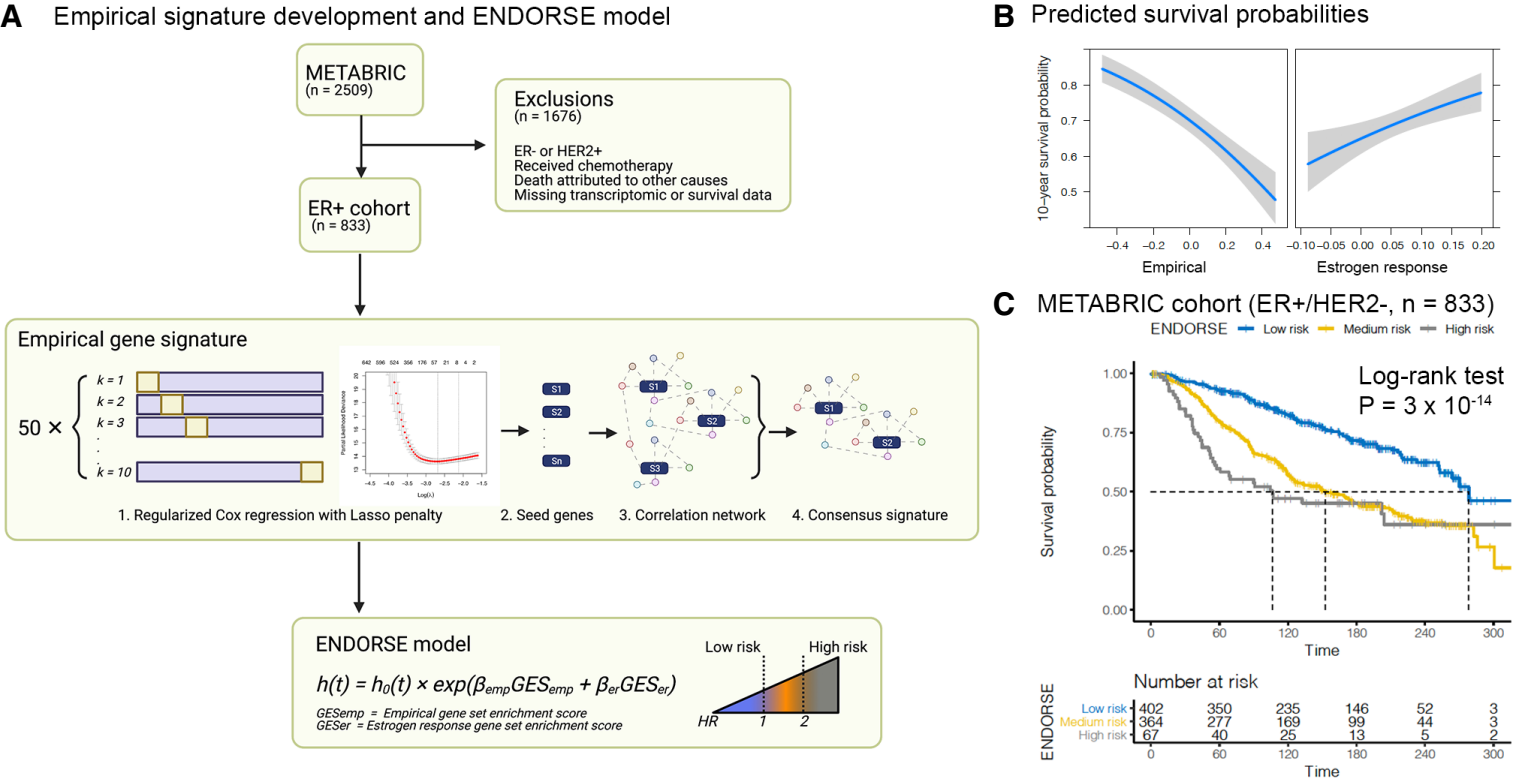
ENDORSE model development in METABRIC Inclusion criteria and schematic of ENDORSE model development. Training samples were selected based on ER status and excluded from the analysis if they were either HER2^+^, received chemotherapy, died due to other causes besides breast cancer, or were missing transcriptomic or survival data. The empirical signature was developed using a repeated cross‐validation analysis framework. Each iteration of the lasso‐regularized proportional hazards model generated a feature set (seed genes) predictive of OS. The seed genes were expanded to a network of intercorrelated genes, and the final empirical signature was defined by identifying a consensus set across all iterations. The two‐feature ENDORSE model was then constructed using the gene set enrichment scores of the empirical signature and estrogen response signature.Predicted 10‐year survival probabilities of the ER^+^/HER2^−^ METABRIC breast cancers (*n* = 833) based on a Cox proportional hazards model of gene signature enrichment scores of the empirical and estrogen response signatures as predictor variables. Grey shaded area indicates 95% confidence intervals of the Cox model predictions.Kaplan–Meier curves and risk tables of METABRIC ER^+^/HER2^−^ tumors stratified by ENDORSE. Dashed lines indicate median survival. *P*‐value were obtained using the log‐rank test for survival curves. The tumors were stratified according to an ENDORSE risk score (hazard ratio) threshold of ≥ 2 to define high‐risk, ≤ 1 as low risk and all other intermediate values as medium risk.

**Figure EV1:**
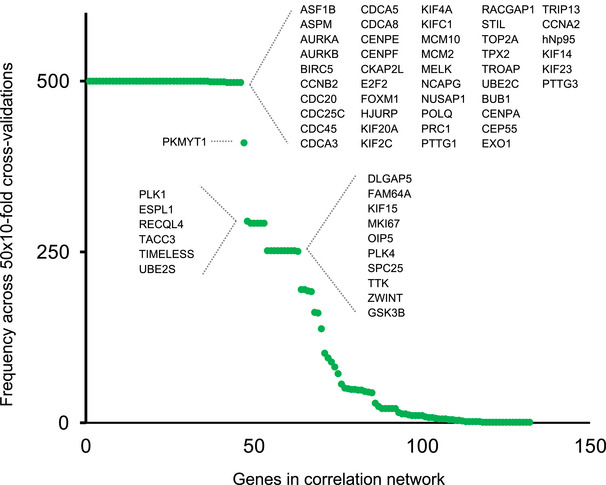
Frequency of genes appearing in the 500 correlation networks generated across 50 × 10‐fold cross‐validations of the LASSO‐regularized Cox proportional hazards model in the METABRIC cohort Y‐axis represents frequency, and X‐axis indicates index of unique genes. Genes on the top left appeared in all 500 cross‐validations, whereas genes at the bottom right appeared only once. Genes appearing in at least 250 networks are marked on the plot.

The objective of this study is to develop a prognostic model for all ER^+^ cancers on endocrine therapy regardless of tumor stage, grade or node status. We developed a two‐component prognostic model for endocrine therapy response using the tumor transcriptomes and long‐term survival outcomes of 833 ER^+^/HER2^−^ tumors that received endocrine therapy (Curtis *et al*, 2012; Pereira *et al*, 2016; Table 1, Fig 1A). About 2 in 5 tumors in this training cohort were node‐positive, while more than a third of the tumors were poorly differentiated, grade 3 tumors (Table 1). The two components included an empirical gene signature modeled on OS (median = 10 years) and a curated gene signature defining response to estrogen (Liberzon *et al*, 2015). Figure 1A outlines the inclusion criteria for the training dataset, method for developing the empirical gene signature and the final ENDORSE model based on the gene set enrichments scores (GES) of the two signatures. The empirical signature was developed by first performing a feature selection on the training dataset using a repeated cross‐validation analysis of a lasso‐regularized proportional hazards model. Each iteration yielded a core set of predictive features that were expanded to a correlation network. The final gene signature was derived from the consensus correlation network, defined as genes appearing in at least 50% of the iterations (Fig EV1, Dataset EV1). In a bivariate Cox proportional hazards model of the training data, the empirical signature was associated with a reduction in survival probability, while the estrogen response signature was associated with improved survival (Fig 1B). The coefficients for the ENDORSE model were calculated using the training cohort, resulting in ENDORSE = 1.54 × (empirical signature GES) – (2.72 × estrogen response GES). The ENDORSE model could also be used to stratify the tumors based on predicted risk. For example, a threshold of ≥ 2‐fold relative risk of death as “high‐risk” and ≤ 1 risk as “low‐risk” resulted in significant difference between the strata in Kaplan–Meier analysis (log‐rank test *P* = 3 × 10^−14^; medium‐risk *P* = 5.43 × 10^−12^, high‐risk *P* = 5.99 × 10^−9^; Fig 1C).

**Table 1:** Training data patient characteristics

Variable	Mean	95% CI	*N* available
Time to event (in months)	135	130–140	833
Events (death due to disease)	0.409	0.376–0.443	833
Age at diagnosis	61.5	60.7–62.3	833
Mutation count	5.55	5.31–5.79	809
Tumor size	24.3	23.4–25.1	828
Tumor stage	1.64	1.59–1.69	634
Stage 0–1 (*n =* 270)			
Stage 2 (*n =* 324)			
Stage > 3 (*n* = 40)			
Tumor Grade	2.29	2.18–2.27	808
Grade 1 (*n =* 103)			
Grade 2 (*n =* 417)			
Grade 3 (*n =* 288)			
Number of positive lymph nodes detected	1.56	1.32–1.79	833
0 (*n =* 491)			
1–3 (*n =* 228)			
4–9 (*n =* 85)			
> 10 (*n* = 29)			

### ENDORSE model performance evaluation with clinical covariates and published signatures in METABRIC

**Figure 2:**
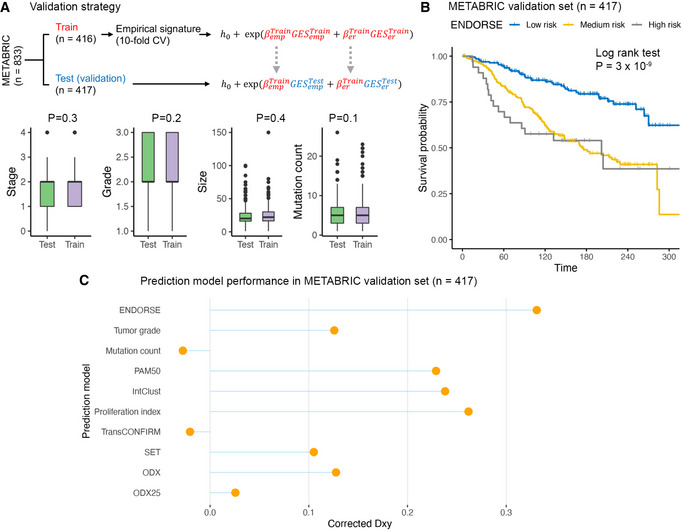
Model evaluation and comparison with other predictors in METABRIC Schematic of validation strategy (top) and boxplots (bottom) comparing key pathological variables between the training and test subsets. The METABRIC cohort was split into a training subset (*n* = 416) and test or validation subset (*n* = 417), with each sample representing an independent biological replicate. The empirical signature was derived using the training split. Then, the ENDORSE model was trained only using the training subset. The coefficients from the training model were used to predict risk in the test subset. The colored boxes in the boxplots display interquartile range with median, while the whiskers show 1.5 × interquartile range. *P*‐values indicate significance of difference in mean from two‐tailed Welch’s *T*‐test.Kaplan–Meier curves and risk tables of the validation subset (*n* = 417) tumors stratified using ENDORSE risk predicted using the training subset model. *P*‐values were obtained using log‐rank test.Lollipop plots displaying corrected Somer’s D_xy_ indices of ENDORSE and various other univariate Cox proportional hazards models in the validation subset (*n* = 417). The corrected D_xy_ indices were calculated using 150‐fold bootstrap resampling of the validation subset.

To evaluate the performance of the ENDORSE model against clinical factors, proliferation index, and published signatures, we created a training and a hold‐out test (validation) subset by performing a 50/50 randomized data split, recalculating the empirical signature using only the training subset and obtaining the ENDORSE model coefficients (Fig 2A). The training and validation subsets had no significant differences in key pathological features, including tumor stage, grade, size and mutation count (Fig 2A). We predicted risk in the held‐out validation subset using the training subset ENDORSE model for performance evaluation. Kaplan–Meier analyses of the validation subset showed a significant difference between the risk strata predicted using the ENDORSE training subset model (log‐rank test *P* = 3 × 10^−9^; medium risk *P* = 6.04 × 10^−8^, high risk *P* = 9.47 × 10^−8^) (Fig 2B).

We performed bootstrap resampling analyses to validate the model in the held‐out dataset and compared with other univariate prognostic models (Fig 2C). First, we compared ENDORSE with clinical factors, such as tumor grade and mutation burden. The ENDORSE model (Somer’s D or D_xy_ = 0.351) performed better than both tumor grade (D_xy_ = 0.129) and mutation count (D_xy_ = −0.027). We also compared the model with a “meta‐PCNA” proliferation index that was reported to capture the prognostic ability of most published signatures of breast cancer (Venet *et al*, 2011; Ramaker *et al*, 2017). Again, the ENDORSE model outperformed the proliferation index (D_xy_ = 0.265), *P* = 4.42 × 10^−5^), indicating its utility over measures of proliferation as a prognostic tool.

Next, we evaluated published prognostic signatures for breast cancers and compared their performance with ENDORSE. These signatures included PAM50, a 50‐gene signature that was previously reported to be a better prognostic tool for ER^+^ breast cancers on endocrine therapy than clinical factors, such as histopathological classification and tumor grade (Nielsen *et al*, 2010). A genomic classifier, IntClust, that was developed by the METABRIC consortium authors and trained on the same training dataset was also included in this comparison (Dawson *et al*, 2013). Both the PAM50 model (D_xy_ = 0.237) and IntClust (D_xy_ = 0.246) models were surpassed by ENDORSE.

**Figure EV2:**
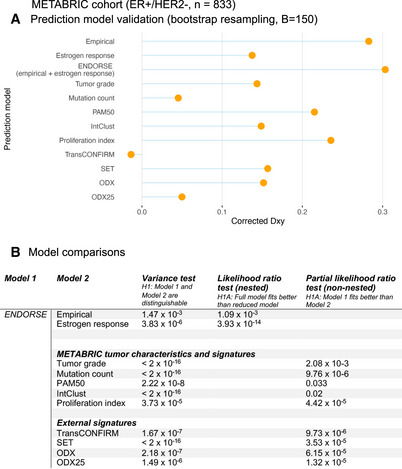
Model evaluation and comparison with other predictors in METABRIC Lollipop plots displaying corrected Somer’s D_xy_ indices of ENDORSE and various other univariate Cox proportional hazards models. The indices were calculated using 150‐fold bootstrap resampling of the training dataset.Table comparing the ENDORSE model with various other univariate Cox models using partial likelihood ratio tests. The comparison between the nested ENDORSE model and its two components was performed using a likelihood ratio test, while other non‐nested univariate models were compared using a partial likelihood ratio test.

Two previous clinical trials evaluating endocrine therapy response in metastatic ER^+^ breast cancers developed prognostic signatures using tumor transcriptomes. The first signature developed in the TransCONFIRM trial included 37 genes that were associated with PFS of advanced ER^+^ breast cancers on fulvestrant (Jeselsohn *et al*, 2016). We replicated the approach described in the study by performing hierarchical clustering of the samples based on the expression levels of the 37 genes and cutting the tree to obtain two clusters. We referred to resultant clusters as the “TransCONFIRM” score. The TransCONFIRM score applied to the METABRIC dataset performed poorly (D_xy_ = −0.023). The second signature (SET ER/PR) was developed using tumor transcriptomes of metastatic ER^+^ breast cancers on endocrine therapy (Sinn *et al*, 2019). This signature included 18 predictive genes that were correlated with *ESR1* or *PGR* expression and normalized using 10 reference transcripts. We implemented the methods described in the original study and referred to the resultant score as “SET.” The SET score (D_xy_ = 0.108) performed better than TransCONFIRM; however, it was also easily outperformed by ENDORSE. Finally, we calculated a surrogate biomarker based on the published formula for the 21‐gene prognostic signature approved for early‐stage, node‐negative ER^+^ breast cancers (Paik *et al*, 2004). We referred to this score as ODX. We also compared a classifier that stratified samples based on the 25^th^ percentile of ODX score as a proxy for the latest risk stratification threshold for this signature (Sparano *et al*, 2018) and referred to this score as ODX25. We found that the ODX model (D_xy_ = 0.127) was comparable to other published signatures like the SET score, but the stratified ODX25 score performed poorly (D_xy_ = 0.021). In addition to the comparisons in the independent validation subset, we also performed similar comparisons between models fitted on the complete METABRIC dataset (Fig EV2A). Using partial likelihood ratio tests, we confirmed the superior performance of the ENDORSE model compared to its components and all other clinical factors and prognostic signatures fitted on the complete dataset (Fig EV2B). These results demonstrate that ENDORSE is a significantly better prognostic model.

**Figure EV3:**
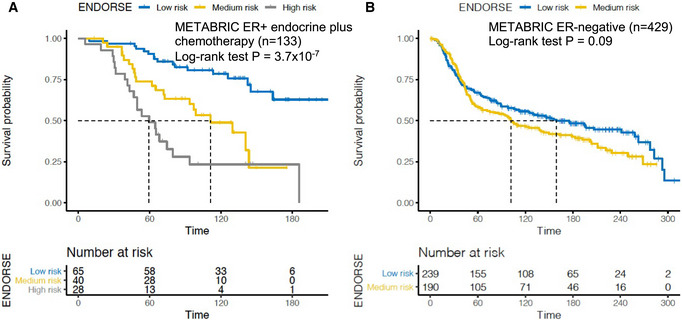
ENDORSE evaluation in ER^+^ tumors receiving chemotherapy and ER‐ tumors A, BKaplan–Meir recurrence‐free survival curves and risk tables of METARBIC cohort ER^+^ patients that received endocrine therapy in (A) combination with chemotherapy (*n* = 133) and (B) METABRIC ER^−^ patients (*n* = 429). In each analysis, the patients were stratified according to ENDORSE predicted risk scores. *P*‐values were obtained using log‐rank tests.

We also applied the ENDORSE risk estimates to stratify 133 METABRIC ER^+^ tumors that received a combination of endocrine therapy and chemotherapy and found a significant difference in the predicted strata (*P* = 3.7 × 10^−7^) (Fig EV3A). On the other hand, applying the ENDORSE model to 429 ER‐negative METABRIC breast cancers as negative control showed no significant difference between the strata (*P* = 0.09, Fig EV3B). These data suggest that the ENDORSE model is specific to the ER^+^ breast cancers and not a general prognostic model to identify aggressive tumors.

### Validation and performance evaluation in independent clinical trial datasets

To test the reproducibility and validate the performance of ENDORSE, we applied the model to the baseline transcriptomes of ER^+^ tumors from four independent clinical trials and compared the ENDORSE‐predicted risk or strata with the outcomes reported in each trial. These independent trials also included the TransCONFIRM and SET ER/PR studies discussed earlier. So, we also compared the performance of TransCONFIRM and SET scores in their respective training datasets and across other independent datasets.

**Figure 3:**
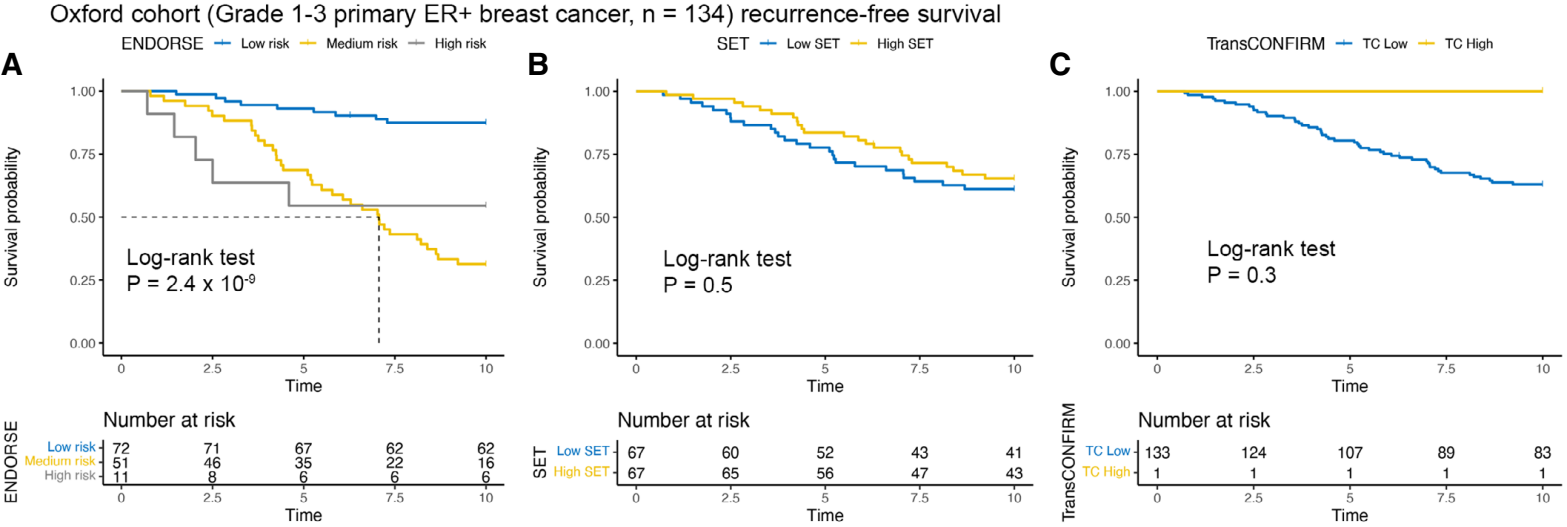
Model validation in Oxford cohort A–CKaplan‐Meir recurrence‐free survival curves and risk tables of Oxford cohort ER^+^ patients (*n* = 134). Dashed lines indicate median survival. The patients were stratified according to (A) ENDORSE (B) SET and (C) TransCONFIRM predicted scores. *P*‐values were obtained using log‐rank test.

First, we evaluated the performance of ENDORSE in the Oxford cohort, an independent validation dataset with similar key histopathological features as the METABRIC dataset. This cohort included 134 ER^+^ breast cancers and about 31% of the tumors were grade III, comparable to the 35% grade III tumors in the METABRIC cohort. The Oxford study reported 10‐year recurrence‐free survival data of the patients. We evaluated the performance of the ENDORSE model and compared it against the SET and TransCONFIRM scores in stratifying the patients. Kaplan–Meier analyses using ENDORSE predicted risk show significant difference in the strata (log‐rank test *P* = 2 × 10^−9^; medium‐risk *P* = 5.23 × 10^−8^, high‐risk *P* = 2.38 × 10^−3^) (Fig 3A). In comparison, both the SET scores (*P* = 0.5) (Fig 3B) and TransCONFIRM scores (*P* = 0.3) (Fig 3C) failed to meaningfully stratify the tumors. Thus, only the ENDORSE model could successfully stratify tumors in a comparable independent dataset.

**Figure 4:**
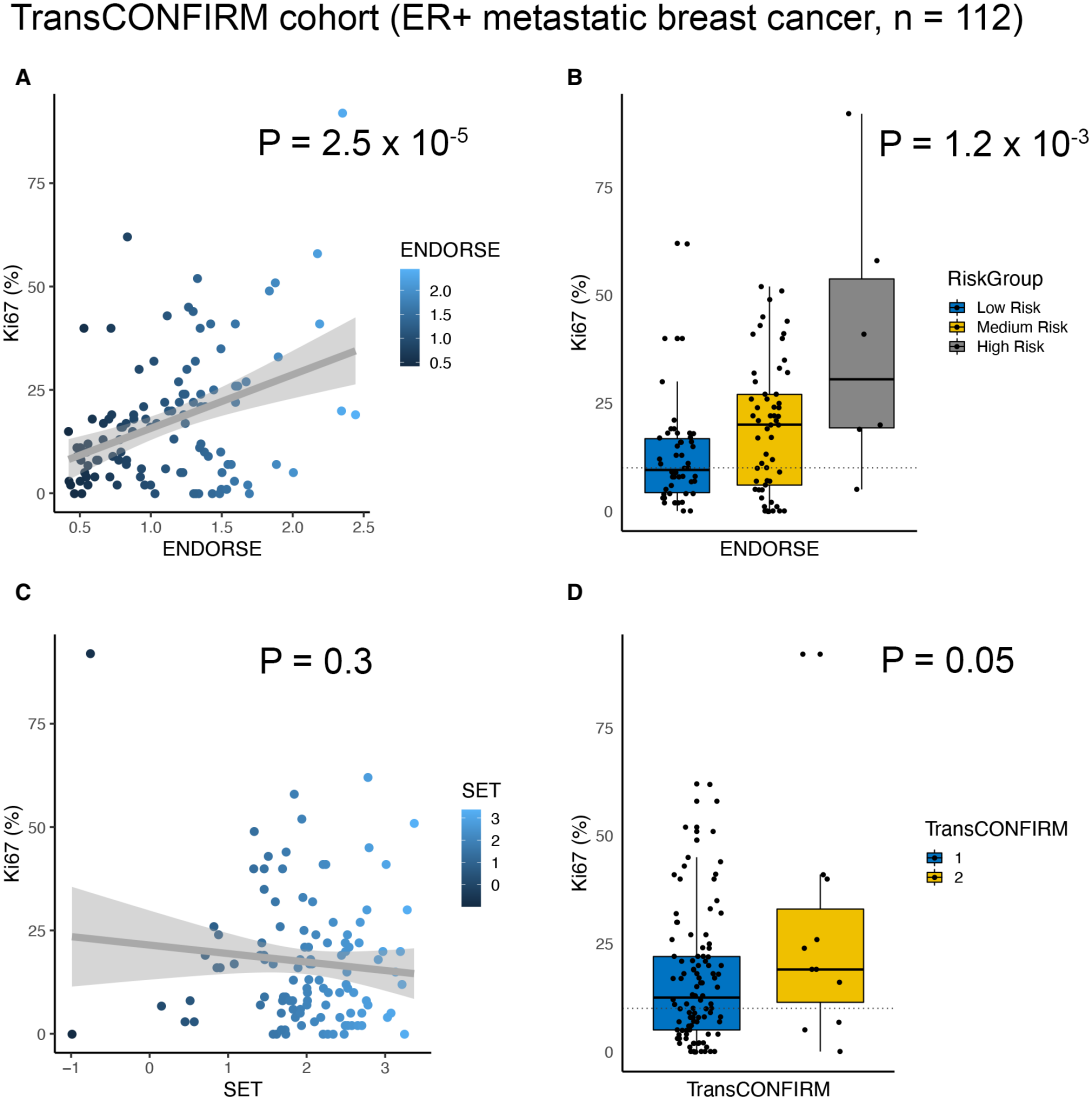
Model validation in TransCONFIRM cohort Scatter plot comparing ENDORSE scores (X‐axis) with TransCONFIRM trial‐reported (*n* = 112) percentage of cells stained positive for Ki67 (Y‐axis). Linear fit is shown as a grey line with shaded region showing 95% confidence intervals (C.I.). *P*‐value indicates significance of the linear fit.Boxplot comparing Ki67% across ENDORSE‐guided patient strata. The colored boxes display interquartile range with median, while the whiskers show 1.5 × interquartile range. *P*‐value indicates significance of the ANOVA model and the horizontal dotted line at 10% indicates threshold of resistance.Scatter plot comparing SET scores (X‐axis) Ki67% (Y‐axis). Linear fit is shown as a grey line with shaded region showing 95% confidence intervals (C.I.). *P*‐value indicates significance of the linear fit.Boxplot comparing Ki67% across TransCONFIRM predicted patient strata. The colored boxes display interquartile range with median, while the whiskers show 1.5 × interquartile range. *P*‐value indicates significance of the ANOVA model and the horizontal dotted line at 10% indicates threshold of resistance.

The TransCONFIRM trial evaluated fulvestrant response in 112 advanced metastatic ER^+^ breast cancers previously treated with an anti‐estrogen (Jeselsohn *et al*, 2016). While the original study developed and evaluated the performance of their 37‐gene signature based on PFS, this survival data was not made available with the publication (the authors did not respond to our requests for this data). However, the study reported the post‐therapy resistant or sensitive states of the tumors based on histopathological staining (Ki67 staining). Therefore, we compared the percentage of cells positive for Ki67 staining reported by in study with risk predictions from ENDORSE and other signatures (Fig 4). The percentage of cells positive of Ki67 was significantly correlated with the ENDORSE estimated risk (*P* = 2.5 × 10^−5^) (Fig 4A), while stratification of the patients based on the risk thresholds also showed a significant difference in Ki67 staining percentage between the strata (ANOVA *P* = 1.2 × 10^−3^; medium‐risk vs. low‐risk *P* = 0.04; high‐risk vs. low‐risk *P* = 1.753 × 10^−4^) (Fig 4B). However, the SET score was not correlated with Ki67 staining (*P* = 0.3) (Fig 4C). The TransCONFIRM score that was developed on this dataset was significant (*P* = 0.05) but performed worse than the ENDORSE score trained on an independent dataset (Fig 4D).

**Figure 5:**
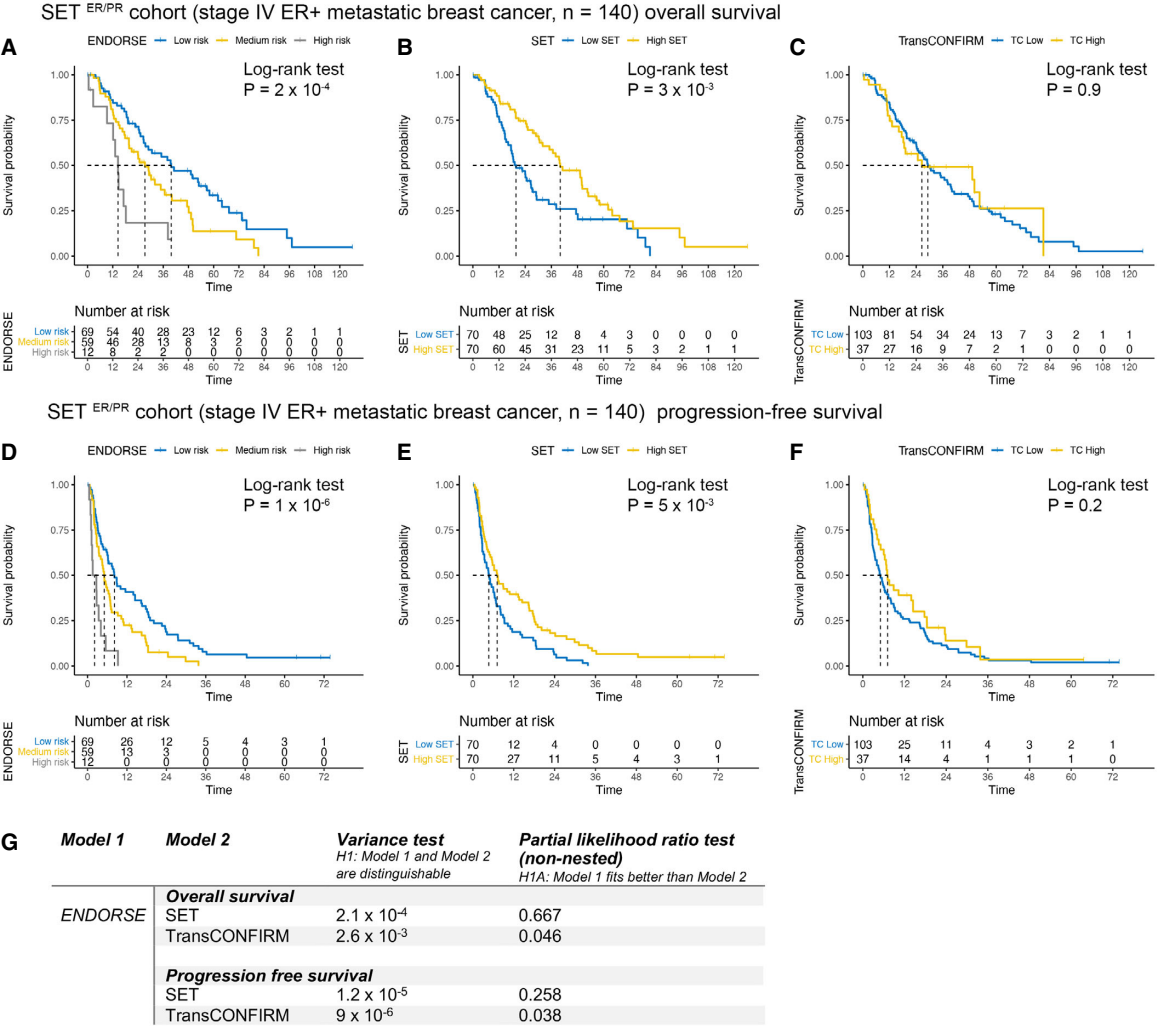
Model validation in SET ER/PR cohort A–COS Kaplan‐Meir curves and risk tables of SET ER/PR patients (*n* = 140). Dashed lines indicate median survival. The patients were stratified according to (A) ENDORSE (B) SET and (C) TransCONFIRM predicted scores. *P*‐values were obtained using log‐rank tests.D–FPFS Kaplan‐Meir curves and risk tables of SET ER/PR patients. Dashed lines indicate median survival The patients were stratified according to (A) ENDORSE (B) SET and (C) TransCONFIRM scores. *P*‐values were obtained using log‐rank tests.GTable comparing the ENDORSE overall and PFS models with SET and TransCONFIRM models using partial likelihood ratio tests for non‐nested Cox models.

Next, we evaluated the performance of the signatures in the SET ER/PR cohort. This clinical trial reported the PFS and OS of 140 stage IV ER^+^ metastatic breast cancers on endocrine therapy. We compared the survival curves of the patients by stratifying them based on the ENDORSE predicted risk, median SET scores, as described in the original study, and the TransCONFIRM score. The stratification based on ENDORSE (log‐rank test *P* = 2 × 10^−4^; medium‐risk *P* = 0.016, high‐risk *P* = 1.88 × 10^−4^) (Fig 5A) and SET (*P* = 3 × 10^−3^) (Fig 5B) scores both resulted in significant differences in the survival curves. (However, the TransCONFIRM score (Fig 5C) was not significant; *P* = 0.9.) Similarly, we observed that ENDORSE (log‐rank test *P* = 1 × 10^−6^; medium‐risk *P* = 0.004, high‐risk *P* = 2.35 × 10^−6^) (Fig 5D) and SET (*P* = 5 × 10^−3^) (Fig 5E) scores both resulted in significant differences in the PFS curves, while TransCONFIRM was not significant (*P* = 0.2) (Fig 5F). Additionally, we compared the model fits using partial likelihood ratio tests (Fig 5G). The SET model that was trained using the same dataset was not a better fit than the ENDORSE model (OS *P* = 0.667, PFS *P* = 0.258). The ENDORSE model was a better fit than the TransCONFIRM model in each case (OS *P* = 0.046, PFS *P* = 0.038).

**Figure 6:**
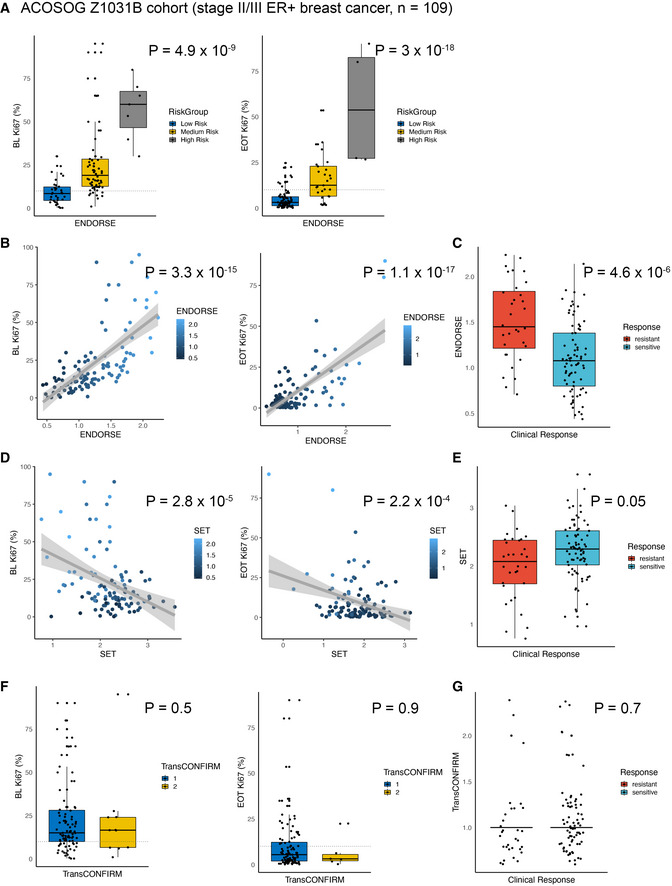
Model validation in ACOSOG Z1031B cohort Boxplots comparing Ki67% at the baseline (left panel) and end of treatment (right panel) across ENDORSE‐predicted patient strata in the ACOSOG Z1031B cohort. The colored boxes display interquartile range with median, while the whiskers show 1.5 × interquartile range. The boxplots represent 109 biological replicates at the baseline (low‐risk *n* = 35, medium‐risk *n* = 67 and high‐risk *n* = 7) or at the end of treatment (low‐risk *n* = 76, medium‐risk *n* = 29 and high‐risk *n* = 4). *P*‐value indicates significance of the ANOVA model and the horizontal dotted line at 10% indicates threshold of resistance.Scatter plot comparing ENDORSE scores (X‐axis) and Ki67% (Y‐axis) at the baseline (left panel) and end of treatment (right panel). Linear fit is shown as a grey line with shaded region showing 95% confidence intervals (C.I.). *P*‐value indicates significance of the linear fit.Boxplots comparing ENDORSE scores between patients grouped by clinical response with 32 resistant and 77 sensitive biological replicates. The colored boxes display interquartile range with median, while the whiskers show 1.5 × interquartile range. *P*‐value indicates significance of the ANOVA model.Scatter plot comparing SET scores (X‐axis) and Ki67% (Y‐axis) at the baseline (left panel) and end of treatment (right panel). Linear fit is shown as a grey line with shaded region showing 95% confidence intervals (C.I.). *P*‐value indicates significance of the linear fit.Boxplots comparing SET scores between patients grouped by clinical response with 32 resistant and 77 sensitive biological replicates. The colored boxes display interquartile range with median, while the whiskers show 1.5 × interquartile range. *P*‐value indicates significance of ANOVA model.Boxplots comparing Ki67% at the baseline (left panel) and end of treatment (right panel) across TransCONFIRM‐predicted patient strata. The colored boxes display interquartile range with median, while the whiskers show 1.5 × interquartile range. The boxplots represent 109 biological replicates at the baseline (cluster 1 *n* = 97, cluster 2 *n* = 12) or at the end of treatment (cluster 1 *n* = 103, cluster 2 *n* = 6). *P*‐value indicates significance of the ANOVA model and the horizontal dotted line at 10% indicates threshold of resistance.Jitter plot comparing TransCONFIRM predictions between patients grouped by clinical response including 32 resistant and 77 sensitive biological replicates with horizontal bars indicating median. *P*‐value indicates significance of the ANOVA model.

In addition to the two metastatic ER^+^ breast cancer trials, we also evaluated the performance of the signatures in the ACOSOG Z1031B clinical trial which evaluated neoadjuvant aromatase inhibitor (AI) treatment in Stage II or III ER^+^ breast cancers (Ellis *et al*, 2017). This study reported percentage of Ki67 staining both at the study baseline and at the end of treatment (2–4 weeks). We compared the percentage of Ki67 positive cells across cancers stratified by the ENDORSE score and found significant difference across the classes at both the baseline (ANOVA *P* = 4.9 × 10^−9^; medium risk vs. low risk *P* = 8.58 × 10^−5^, high risk vs. low risk *P* = 0) and at the end of treatment (ANOVA *P* = 3 × 10^−18^; medium risk vs. low risk *P* = 1.6 × 10^−6^, high risk vs. low risk *P* = 0) (Fig 6A). Similarly, the continuous ENDORSE scores were significantly correlated with both the baseline (*P* = 3.3 × 10^−15^) and end of treatment (*P* = 1.1 × 10^−17^) Ki67 percentage (Fig 6B). The ENDORSE scores were also significantly higher in the tumors that were classified as resistant based on clinical response (*P* = 4.6 × 10^−6^) (Fig 6C). In this cohort, the SET score was also significantly correlated with Ki67 percentage at the baseline (*P* = 2.8 × 10^−5^) and end of treatment (*P* = 2.2 × 10^−4^) (Fig 6D), with a significant difference in the SET scores between the resistant and sensitive tumors (*P* = 0.05) (Fig 6E). The transCONFIRM scores were not significant at the baseline (*P* = 0.5) and end of treatment (*P* = 0.9) (Fig 6F) or between resistant and sensitive tumors (*P* = 0.7) (Fig 6G).

### Common pathway phenotypes and somatic alterations enriched in high‐risk tumors

**Figure 7:**
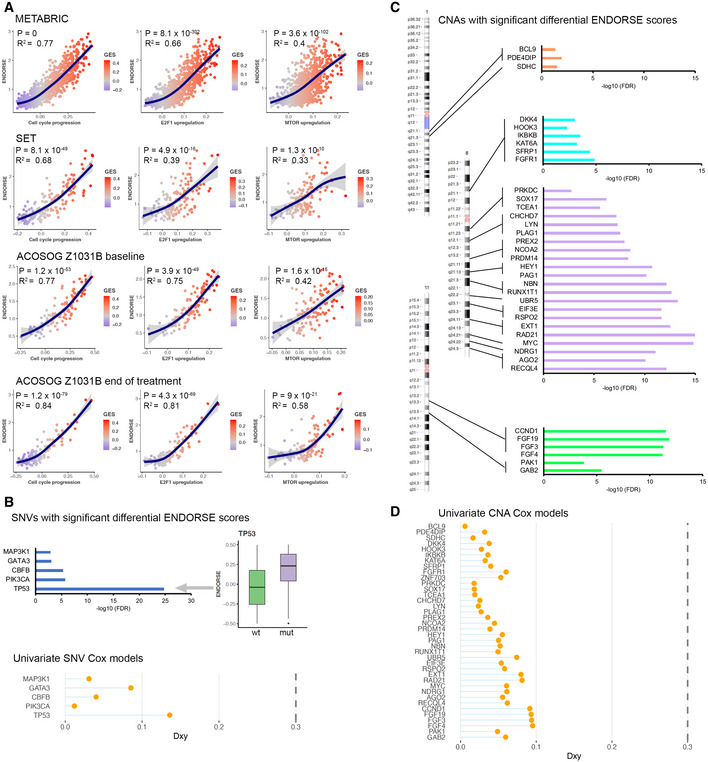
Biology of the high‐risk tumors Scatter plots displaying gene set enrichment scores (GES) of key pathways (X‐axis) and ENDORSE scores (Y‐axis) in the METABRIC ER^+^ cohort (*n* = 833). The cell cycle progression panel represents the hallmark G2 M checkpoint signature, the E2F1 upregulation panel represents E2F1_UP.V1_UP oncogenic (C6) signature and the MTOR upregulation panel represents MTOR_UP.V1_UP oncogenic (C6) signature. Blue lines with shading indicate generalized additive model fits with 95% C.I., with *R* ^2^ and *P*‐values of the significant of the fit annotated on the panels.Bar plots showing false discovery rate‐adjusted *P*‐values from the ANOVA analysis of ENDORSE scores with mutation status as the grouping variable. The boxplot on the right shows difference in the ENDORSE scores between *TP53* mutant (*n* = 159) and wild type tumors (*n* = 674). The colored boxes display interquartile range with median, while the whiskers show 1.5 × interquartile range, with each sample representing a biological replicate. The lollipop plot below shows Somer’s D_xy_ of the univariate Cox models for the SNVs, with the vertical dotted line indicating D_xy_ of the ENDORSE model.Ideograms showing mapped regions with copy number gains that are significant in ANOVA analysis of ENDORSE scores with copy number gain status as the grouping variables. Bar plots on the right show false discovery rate‐adjusted *P*‐values from the ANOVA analysis.Lollipop plot showing Somer’s D_xy_ of the univariate Cox models for the copy number gains, with the vertical dotted line indicating D_xy_ of the ENDORSE model.

We analyzed the pathway phenotypes enriched in each dataset to identify potential mechanisms that defined the high‐risk tumors. First, we calculated the GES for 50 hallmark, 4,690 curated, and 189 oncogenic signatures from the METABRIC transcriptomes and fitted a generalized additive model for ENDORSE scores with each signature as the predictor (Datasets EV2–EV4). We found multiple hallmark signatures and oncogenic pathways to be significantly associated with the ENDORSE scores (Datasets EV2–EV4). Key enriched hallmark signatures included MTOR signaling (*P* = 1.03 × 10^−72^) and MYC targets (v2, *P* = 2.66 × 10^‐83^), while key oncogenic signatures included gain in E2F1 target expression (*P* = 8.06 × 10^−302^) and loss of RB1 activity via p107 and p130 (*P* = 9.51 × 10^−137^, 1.31 × 10^−67^) (Datasets EV2 and EV4). Next, we calculated the GES for the hallmark and oncogenic signatures in the three validation datasets (Datasets EV5–EV10). We observed that pathways associated with cell‐cycle progression and proliferation, along with signatures for the loss of RB1 activity and activation of the PI3K/AKT/MTOR signaling pathways, were generally enriched across the METABRIC and all the three validation datasets (Fig 6A). Similar to the training dataset, we also found gain in cell cycle progression along with MTOR signaling and E2F1 target expression to be associated with high ENDORSE scores across all datasets (Fig 7A, Datasets EV5–EV10). The commonality of the signatures enriched across different datasets suggested that similar underlying phenotypes were acquired by the high‐risk tumors.

**Figure EV4:**
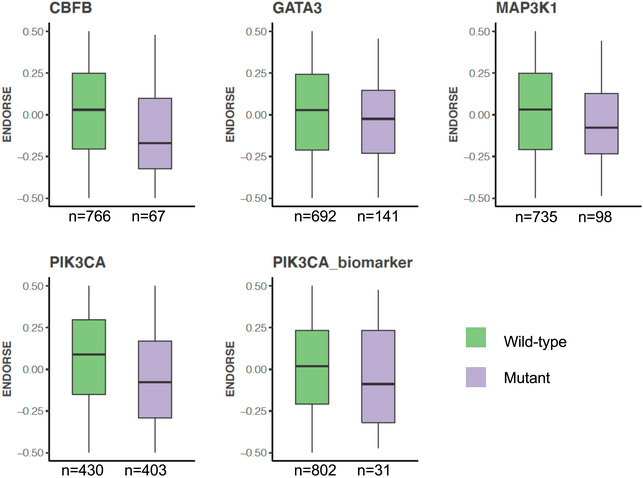
Somatic variants associated with ENDORSE scores Boxplots showing ENDORSE predicted risk scores in METABRIC that were significantly different in mean values (false discovery rate‐adjusted *P* < 0.05) in samples grouped by mutation status of cancer genes. The colored boxes display interquartile range with median, while the whiskers show 1.5 × interquartile range, with each sample representing a biological replicate. The numbers of wild type and mutated tumors serving as biological replicates are annotated below the boxplots for each gene.

We also analyzed the association between gene‐level somatic mutations, including non‐synonymous single‐nucleotide variants (SNV) and copy number alterations, with the ENDORSE scores of the METABRIC ER^+^ tumors. We found a statistically significant association (FDR < 0.05) between the ENDORSE scores and SNVs of only five genes (Fig 7B, Dataset EV11). While *PIK3CA* mutations were found in ~50% of all tumors, we found that ENDORSE scores were not significantly higher in tumors with non‐synonymous *PIK3CA* variants or activating *PIK3CA* variants that guide the use of PI3K inhibitors (Fig EV4). Of the five significant genes, only tumors with *TP53* mutations showed a significantly higher ENDORSE score (Fig 7B). We then performed bootstrap analyses with the univariate Cox models of the significant genes and found that none of the SNV Cox models performed better than the ENDORSE model (Fig 7B).

**Figure EV5:**
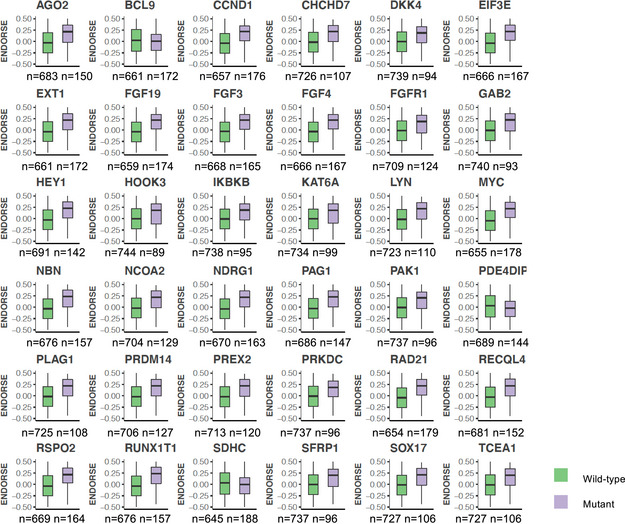
Copy number amplifications associated with ENDORSE scores Boxplots showing ENDORSE predicted risk scores in METABRIC that were significantly different in mean values (false discovery rate‐adjusted *P* < 0.05) in samples grouped by copy number amplification status of cancer genes. The colored boxes display interquartile range with median, while the whiskers show 1.5 × interquartile range. The numbers of wild type (no gain) and mutated (copy number amplified) tumors serving as independent biological replicates are annotated below the boxplots for each gene.

Several gene‐level amplifications were also associated with significant differences in ENDORSE scores (Figs 7C and EV5). Interestingly, the significant amplifications were localized at chromosome 1q, 8p, 8q, or 11q, suggesting that different genetic alterations affecting a recurring set of loci may be correlated with the emergence of resistance in the high‐risk tumors (Fig 7C, Dataset EV12). Like the univariate SNV models above, the univariate copy number alteration models also performed poorly when compared to the ENDORSE model in bootstrap resampling analyses (Fig 7D).

Finally, we evaluated whether the ENDORSE model reflects different key phenotypes associated with early (within 5 years) vs. late (beyond 5 years) mortality. For patients with an ENDORSE risk > 1, we found that tumors associated with late mortality showed elevated estrogen response signatures, whereas tumors linked with early mortality showed enrichment of epithelial to mesenchymal transition, stemness, angiogenesis, and related signaling pathways that are known to contribute to these phenotypes including TGFB, YAP, MEK, and AKT pathways (Datasets EV13–EV15).

## Discussion

Breast cancers are classified as ER^+^ based on a broad criterion of positive immunohistochemical staining of 1–100% of cell nuclei for the estrogen receptor (Rugo *et al*, 2016; Allison *et al*, 2020). However, ER^+^ tumors are heterogeneous, both in terms of dependence on estrogen signaling for growth and survival and intrinsic or acquired resistance to endocrine therapy (Musgrove & Sutherland, 2009; Spoerke *et al*, 2016). Therefore, optimal clinical management of each ER^+^ breast cancer depends on accurate prediction of response to endocrine therapy and selection of companions for endocrine therapy. Several genomic tests are available for classifying breast cancers into molecular subtypes (Parker *et al*, 2009) or assessing the likelihood of benefit from chemotherapy in early‐stage, node‐negative ER^+^ breast cancers (Paik *et al*, 2004; Cardoso *et al*, 2016). Results from the MINDACT and TAILORx studies (Cardoso *et al*, 2016; Sparano *et al*, 2018) show that it is possible for node‐negative, early‐stage breast cancers to safely waive additional chemotherapy if they are predicted to be at a low risk of recurrence based on genomic signatures. However, these tests have not been proved to be useful in the advanced and metastatic ER^+^ breast cancer setting. The default primary treatment for advanced ER^+^ breast cancer remains endocrine therapy, despite proven benefits from add‐on targeted therapy or potential switch to chemotherapy. Therefore, the key challenge in advanced ER^+^ breast cancer is to stratify patients that will likely benefit from continued endocrine therapy and patients that are likely resistant to single‐agent endocrine therapy and will benefit from selecting a different treatment strategy (Hart *et al*, 2015).

To address this challenge, we have developed a new prognostic model to predict endocrine response in advanced ER^+^ breast cancers. We developed our model using invasive tumors from the METABRIC study that were ER^+^ and included node‐positive, high‐grade tumors. Our model addressed several challenges associated with the development of genomic biomarkers. Since the number of available features to train the genomic models tend to be much larger than the number of available samples (*P* >> *n*), it is quite easy to create complex prediction models that contain many predictor variables. Often, such models perform very well in the training datasets, but the performance cannot be replicated in independent test datasets due to overfitting. Several approaches have been proposed to address this issue. Broadly, these can be classified into unsupervised and supervised approaches. The unsupervised approach typically relies on grouping or clustering the samples based on the similarity of gene expression profiles, followed by analysis of association with survival outcomes (Sotiriou *et al*, 2003). Alternatively, a supervised approach is to perform dimensionality reduction prior to modeling the survival outcome or drug response using univariate or multivariate models (Paul *et al*, 2008). Our model utilized the later strategy by using a regularized Cox model for feature selection, effectively reducing the dimensionality of the gene expression data. We further collapsed the genes into a signature and parameterized the final Cox model on the GES of the signatures. The rank‐based approach to calculate GES also helped mitigate issues associated with batch effects and differences in methods for transcriptome profiling. We performed an extensive performance evaluation of our model against other published signatures and clinical factors. Consistently, we found that the ENDORSE model was a better predictor than all other models in the training dataset (Fig 2). Moreover, ENDORSE clearly outperformed all other published signatures when they were applied to external validation datasets (Figs 3, 4, 5, 6). Our results show that ENDORSE is a highly accurate and reproducible model that outperforms current approaches to predict endocrine response in metastatic ER^+^ breast cancer.

We also explored the biology of the ER^+^ tumors to identify possible mechanisms that are commonly shared by high‐risk tumors. We found that high‐risk tumors showed a consistent enrichment of pathways‐associated cell cycle progression and gain of PI3K/MTOR signaling pathways (Fig 7A). In addition, we observed consistent gain of the E2F1 signature, which may be associated with metastatic progression of breast cancers (Hollern *et al*, 2014, 2019). We also observed a loss of Rb1 activity, which has been associated with therapeutic resistance in ER^+^ breast cancers (Bosco *et al*, 2007; Witkiewicz & Knudsen, 2014). In the METABRIC cohort, tumors with early mortality (within 5 years of sample collection) showed consistent enrichment of pathways that are known to contribute to epithelial to mesenchymal transition, angiogenesis, and stemness. Enriched pathways, including TGF‐beta (Band & Laiho, 2011) and YAP (Ma *et al*, 2022), have been suggested to directly crosstalk and repress the estrogen receptor signaling pathway. Concurrently, MEK (Fujii *et al*, 2011) and PI3K/AKT/MTOR (Ciruelos Gil, 2014) have been implicated as key primary resistance mechanisms in endocrine‐resistant ER^+^ breast cancers. In contrast, tumors with late mortality did not show enrichment of specific signaling pathways, suggesting that diverse resistance mechanisms may evolve long term and contribute to the late relapse of these tumors. Characterizing the heterogeneity in late relapse tumors would be necessary to understand the diverse mechanisms that lead to the evolution of resistance in these tumors (Zardavas *et al*, 2015).

In addition to common pathway phenotypes shared across high‐risk tumors, mutations in the *TP53* tumor suppressor genes were also significant (Fig 7B). Loss‐of‐function *TP53* variants have long been associated with aggressiveness and chemotherapeutic resistance in hormone‐receptor‐negative breast cancers (Cattoretti *et al*, 1988; Elledge *et al*, 1993). However, recent studies show that even though *TP53* mutations are infrequent in ER^+^ breast cancers, they have similar negative impact on patient outcome as hormone‐receptor‐negative breast cancers (Ungerleider *et al*, 2018). We also found recurrent copy number gains at chromosomes 8 and 11 to be associated with high‐risk tumors (Fig 7C). Amplifications at these loci have been previously associated with aggressive and drug‐resistant cancers and included several oncogenes such as *MYC*, *CCND1*, and multiple fibroblast growth factors (Lundgren *et al*, 2008; Baslan *et al*, 2020). The survival models based on genomic alterations were clearly outperformed by ENDORSE; however, the recurrent nature of these alterations in high‐risk tumors suggests that further studies to investigate their role in promoting endocrine resistance are warranted.

Drugs that target CDK4/6 to inhibit cell cycle activation (Hortobagyi *et al*, 2016), PI3K‐inhibitors that target tumor with activating *PIK3CA* mutations (André *et al*, 2019), and MTOR‐inhibitors that prevent the activation of MTOR signaling and cell proliferation (Baselga *et al*, 2012) have been studied and approved for the treatment of advanced ER^+^ breast cancers in combination with endocrine therapy. However, patients first advance on primary endocrine therapy, with or without additional CDK4/6 inhibitors before they are stratified in a different treatment arm. Therefore, identifying high‐risk tumors with the ENDORSE model prior to first‐line administration of single‐agent endocrine therapy could help identify which cancers may be better suited for an add‐on regimen or switching to chemotherapy. Thus, future clinical trials applying the ENDORSE model may benefit from early and accurate prediction of endocrine response in advanced, metastatic ER^+^ breast cancers. This could ultimately help prolong the survival of patients by stratifying into more appropriate treatment groups.

## Materials and Methods

### Data retrieval and pre‐processing

METABRIC microarray gene expression, mutations, copy number alterations, and clinical annotations survival data were retrieved using cBioPortal for cancer genomics (https://www.cbioportal.org/study/summary?id=brca_metabric) (Gao *et al*, 2013; Data ref: Gao *et al*, 2013). Independent validation datasets used in this study were: SET ER/PR microarray gene expression: Gene Expression Omnibus, accession number: GSE124647 (https://www.ncbi.nlm.nih.gov/geo/query/acc.cgi?acc=GSE124647). (Sinn *et al*, 2019; Data ref: Sinn *et al*, 2019), TransCONFIRM microarray gene expression: Gene Expression Omnibus: accession number: GSE76040 (https://www.ncbi.nlm.nih.gov/geo/query/acc.cgi?acc=GSE76040). (Jeselsohn *et al*, 2016; Data ref: Jeselsohn *et al*, 2016), ACOSOG Z1031B microarray gene expression: Gene Expression Omnibus, accession number: GSE87411 (https://www.ncbi.nlm.nih.gov/geo/query/acc.cgi?acc=GSE87411). (Ellis *et al*, 2017; Data ref: Ellis *et al*, 2017), and Oxford cohort microarray gene expression: Gene Expression Omnibus, accession number: GSE22219 (https://www.ncbi.nlm.nih.gov/geo/query/acc.cgi?acc=GSE22219). (Buffa *et al*, 2011; Data ref: Buffa *et al*, 2011).

To ensure that gene expression datasets generated using diverse platforms are comparable and appropriate for predictive analyses, we uniformly applied the following pre‐processing steps on all datasets. First, we removed genes with zero variance from each dataset and summarized genes with multiple probes by mean expression. Next, we log_2_ transformed the expression levels, unless the data was already provided as log‐transformed values. Finally, we scaled and standardized the expression levels such that each gene had a mean expression level of zero and a standard deviation of one.

### Inclusion criteria for METABRIC training cohort

The METABRIC cohort contained a total of 2,509 samples. Samples that met all of the following criteria were included in the training cohort: patients that were ER‐positive and HER2‐negative based on immunohistochemistry, patients that received hormone therapy but did not receive additional chemotherapy, patients that were either alive or died due to the disease and no other causes, and patients with complete survival and transcriptomic data. After filtering, 833 samples were retained for model construction.

### Empirical signature and ENDORSE model construction

The empirical gene signature was developed using a LASSO‐regularized Cox proportional hazards models, with OS as the outcome variable (Tibshirani, 1996). The hazard function in the Cox model is defined as:

$$h_{i}\left(t\right)=h_{0}\left(t\right)\exp\left({\beta \text{x}}_{i}^{T}\right)$$

where X is a set of predictive gene expression features and h_0_ is an arbitrary baseline hazard function. The coefficient (β) for each predictor in the model can be estimated by maximizing the partial likelihood function L(β), defined as:

$$L\left(\beta \right)=\prod_{i}\frac{\exp\left({\beta \text{x}}_{j\left(i\right)}^{T}\right)}{\sum_{I\in R_{i}}\exp\left({\beta \text{x}}_{j}^{T}\right)}$$

where R_i_ is the set of indices of observations failing (events) at time t_i_. In the LASSO Cox model, the regularized coefficient is obtained by adding a penalty parameter λ to the log of the likelihood function.

$$\hat{\beta }=\min‐\frac{1}{N}l\left(\beta \right)+\lambda {\left|\left|\beta \right|\right|}_{1}$$

where l(β) = log L(β). The λ penalty parameter was determined using 10‐fold cross‐validation implemented in R package glmnet (Friedman *et al*, 2010; Simon *et al*, 2011). The optimal λ minimized model deviance.

We applied the model in a repeated (50 × 10‐fold) cross‐validation framework. In each iteration, a set of “seed genes” or features with positive coefficients in the regularized Cox model at a λ equal to one standard error from the minimum model deviance were identified. The seed genes were expanded to a redundant correlation network by adding all genes in the training transcriptome dataset that had Pearson’s correlation > 0.75 with any of the seed genes. Across all iterations, we identified the common set of features that were present in at least 50% of the correlation networks and defined this set of features as the empirical signature.

The ENDORSE model was defined as the hazard’s ratio of the Cox proportional hazards model fitted on OS data of the training cohort with two components: GES for the empirical gene signature and GES for the hallmark estrogen early response signature.

$$h\left(t\right)=h_{0}\left(t\right)\times \exp\left(\beta_{\mathrm{emp}}GES_{\mathrm{emp}}+\beta_{\mathrm{er}}GES_{\mathrm{er}}\right).$$

where *emp* represents the empirical signature and *er* represents the estrogen response signature.

For each signature, the GES were calculated for individual samples using the GSVA package for R (Hänzelmann *et al*, 2013) using the ssGSEA method (Barbie *et al*, 2009). The ssGSEA method is especially well suited for calculating GES from data from diverse platforms, as the method uses empirical cumulative distribution functions of the ranked values of probe or gene expression to calculate enrichment scores. By using ranked expression values, this method mitigates challenges encountered with applying predictive models on datasets with vastly different distribution profiles than the training data. The final parameters for the ENDORSE model were obtained by fitting the model to the full training cohort of 833 samples, resulting in β_emp_ = 1.54 and β_er_ = −2.72.

### Models based on external signatures and clinical factors

Clinical features such as tumor grade and mutation count, along with scores from PAM50 and IntClust analyses, were obtained directly from the METABRIC clinical annotations accompanying the transcriptome data and were directly utilized in univariate Cox models. Proliferation index based on the metaPCNA signature was calculated using the R‐package ProliferativeIndex (Ramaker *et al*, 2017). We replicated the signatures and algorithms developed in the TransCONFIRM, SET ER/PR, and 21‐gene prognostic signature studies by following the methods described in the respective studies. The TransCONFIRM signature composed of 37 genes was implemented by performing hierarchical clustering of the gene expression data using these genes and cutting the tree (*k* = 2) to stratify samples in high or low TransCONFIRM score categories. The SET signature was implemented by calculating (the average expression of the 18‐genes in the signature) − (the expression of 10 house‐keeping genes) + 2. The 21‐gene signature (ODX) score was calculated by following the unscaled risk score calculation reported by the study. *BAG1* transcript was missing from the METABRIC cohort and was not included in the unscaled score calculation. Since this transcript was uniformly missing on all samples, the relative risk scores could be compared across the samples.

### ENDORSE model performance evaluation in METABRIC

The predictive ability of ENDORSE and various other models were evaluated in the METABRIC training dataset using two approaches. In the first approach, we split the METABRIC cohort (50/50) into a training and hold‐out test or validation subset. We applied the LASSO‐regularized Cox proportional hazards model on only the training subset to obtain an empirical signature using 10‐fold cross‐validation. Next, we calculated GES of the training subset‐derived empirical signature and the estrogen response signature and fit a Cox proportional hazards model on the OS data of the training subset. Using this model, we predicted the OS of the held‐out test subset. To compare the performance of the model against other independent signatures and clinical features associated with each sample, we performed a bootstrap resampling analysis of the Cox regression models. The resampling was repeated 150 times for each model and a Somer’s D_xy_ rank correlation was calculated in each repeat. A final bias‐corrected index of Somer’s D_xy_ was obtained as a measure of the model’s predictive ability. The bootstrap resampling and calculations of the Somer’s D_xy_ were performed using the R package “rms.” Models based on SNVs and CNAs significantly associated with ENDORSE scores were also evaluated by obtaining Somer’s D_xy_ rank correlation metric of the univariate Cox model. In the second approach, we calculated the ENDORSE scores of the full cohort and compared each of the external signatures and clinical feature models using Vuong’s (Vuong, 1989) partial likelihood ratio test for non‐nested Cox regression models. The non‐nested partial likelihood ration tests were implemented using the R package “nonnestcox” (https://github.com/thomashielscher/nonnestcox/). The individual components of the ENDORSE model were compared to the full model using likelihood ratio tests for nested Cox models.

### Model validation in independent datasets

We compared the predictive performance of ENDORSE in multiple independent datasets. First, we integrated the training (METABRIC) and test (independent validation) datasets to perform batch correction using the ComBat function of the R package “sva” (Leek *et al*, 2012). Next, we calculated the GES for the ENDORSE signatures in the batch corrected METABRIC and independent validation gene expression datasets. Then, the parameters of the ENDORSE Cox model were calculated on the batch‐corrected training dataset with the OS information as the response variable. Finally, the parameterized Cox model was applied to the independent validation dataset to obtain a predicted risk score.

In case of the Oxford and SET ER/PR cohort, we used the predicted ENDORSE risk scores to stratify the patients into risk categories, with an ENDORSE score ≥ 2 representing the high‐risk group, ≤ 1 representing the low‐risk group, and other intermediate values representing the medium risk group. We compared the significance of stratification of recurrence‐free survival curves in the Oxford cohort and both OS and PFS curves in the SET ER/PR cohort based on ENDORSE, SET, and TransCONFIRM scores using log‐rank tests. Further, we compared individual strata using Cox models and partial likelihood ratio tests to compare non‐nested Cox models.

For the TransCONFIRM and ACOSOG cohorts, we compared the ENDORSE (with an ENDORSE score ≥ 2 representing the high‐risk group, ≤ 1 representing the low‐risk group and other intermediate values representing the medium risk group), SET, and TransCONFIRM predictions with reported clinical variables, such as percentage of cells positive for Ki67 at the end of treatment and clinical outcomes using generalized linear models for continuous outcome variables or one‐way ANOVA analysis for categorical outcomes. *Post‐hoc* analyses comparing significance of difference in means between different ENDORSE risk groups were obtained using Tukey’s HSD test.

### Biological features associated with ENDORSE scores

To determine the possible biological mechanisms associated with the emergence of endocrine resistance and high ENDORSE risk scores, we evaluated the enrichment scores of various biological pathway and oncogenic signatures across the training and independent validation cohorts. We used the ssGSEA method to obtain GES for hallmark, curated (C2), and oncogenic signature (C6) gene sets from the molecular signatures database (Liberzon *et al*, 2011). For each signature, we fitted a generalized additive model against the predicted ENDORSE score to obtain significance of the fit, R^2^, and proportion of variance explained by the model. None of the curated signatures were significant in the METABRIC analyses and were excluded from further consideration in the independent validation datasets. Pathways enriched in early mortality (< 5 years OS) vs. late mortality (> 5 years OS) ER^+^ METABRIC tumors with an ENDORSE risk score > 1 were determined using Welch’s *t*‐test.

Gene‐level somatic SNV and CNV analyses were performed using data reported by the METABRIC study. SNVs were retained based on a mutation frequency of ≥ 5 across all samples and limited to genes that are known cancer‐related genes, obtained from the Catalogue of Somatic Mutations in Cancer (COSMIC) cancer gene census (Sondka *et al*, 2018; Tate *et al*, 2019). Pathogenic *PIK3CA* variants associated with PI3K inhibitor sensitivity were obtained from the drug labels for alpelisib based on the SOLAR1 clinical trial (André *et al*, 2019). Significant SNVs and CNVs were obtained using a one‐way ANOVA analysis of the ENDORSE scores with mutation status as the factor.

## Author contributions

**Aritro Nath:** Conceptualization; Data curation; Software; Formal analysis; Validation; Investigation; Visualization; Methodology; Writing—original draft; Writing—review and editing. **Adam L Cohen:** Conceptualization; Resources; Supervision; Funding acquisition; Investigation; Methodology; Project administration; Writing—review and editing. **Andrea H Bild:** Conceptualization; Resources; Supervision; Funding acquisition; Investigation; Methodology; Project administration; Writing—review and editing.

In addition to the CRediT author contributions listed above, the contributions in detail are:

Conceptualization, Methodology, Investigation—AN, ALC, AHB; Data Curation, Formal Analysis, Software, Visualization, Validation—AN; Funding Acquisition, Resources, Supervision, Project Administration—ALC, AHB; Writing—original draft—AN; Writing—review & editing—AN, ALC, AHB.

## Disclosure and competing interests statement

The authors AN and AHB are listed as co‐inventors on a patent application (filed, pending) covering the ENDORSE biomarker model described in this manuscript.

## Supporting information



Expanded View Figures PDF

Dataset EV1

Dataset EV2

Dataset EV3

Dataset EV4

Dataset EV5

Dataset EV6

Dataset EV7

Dataset EV8

Dataset EV9

Dataset EV10

Dataset EV11

Dataset EV12

Dataset EV13

Dataset EV14

Dataset EV15

## Data Availability

Scripts for developing ENDORSE, validation using METABRIC and other independent clinical trials datasets, and analysis to determine significant pathways and somatic variants associated with high ENDORSE score tumors are available on GitHub: https://github.com/aritronath/ENDORSE. The gene set components of the ENDORSE model and training datasets are also available on the GitHub repository under releases.
