# Supplementary material for: ENDORSE: a prognostic model for endocrine therapy in estrogen‐receptor‐positive breast cancers
Source: Mol Syst Biol. 2022 Jun 7;18(6):e10558. doi: 10.15252/msb.202110558 (PMC9172932; doi:10.15252/msb.202110558)
Supplement: Supplementary file 1 — Expanded View Figures PDF [file MSB-18-e10558-s015.pdf]

Expanded View Figures

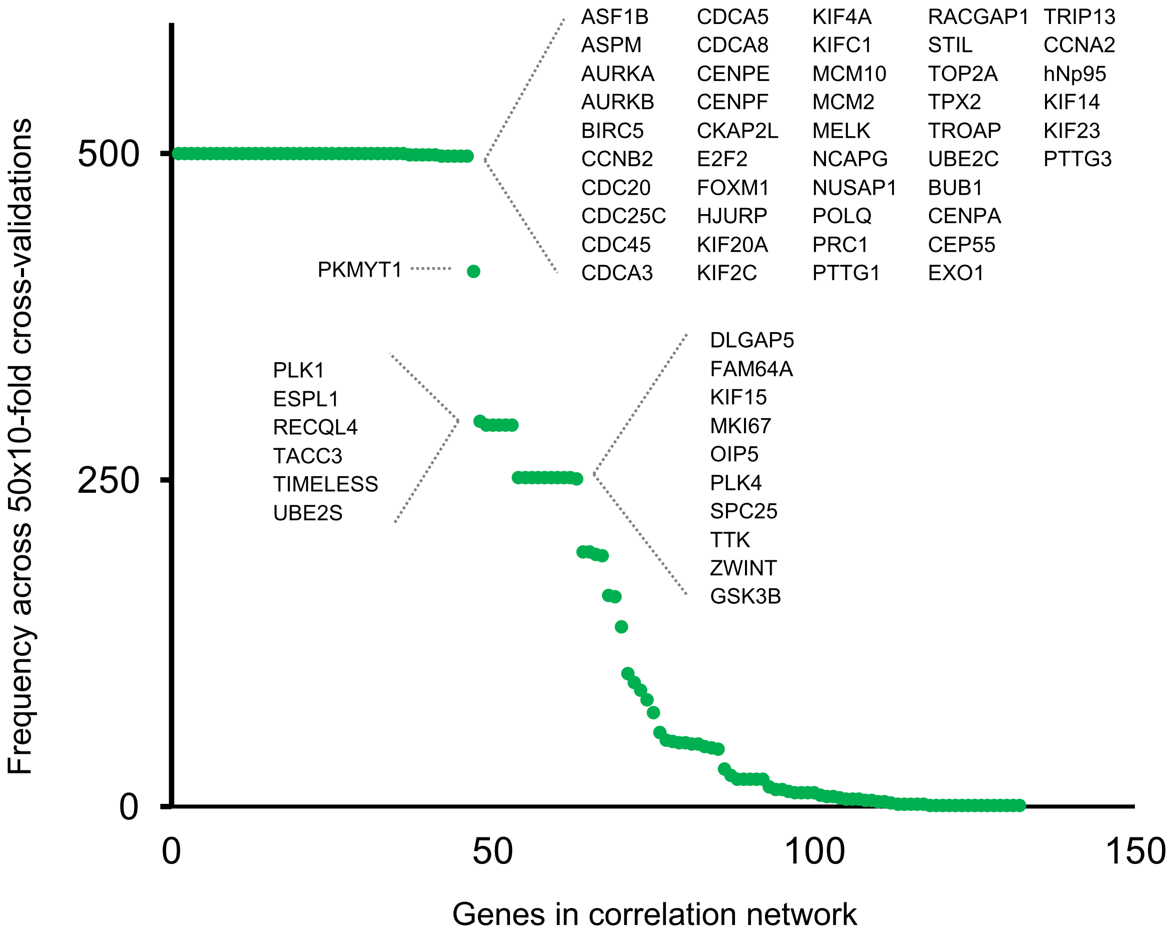

Figure EV1. Frequency of genes appearing in the 500 correlation networks generated across 50 × 10-fold cross-validations of the LASSO-regularized Cox proportional hazards model in the METABRIC cohort.

Y-axis represents frequency, and X-axis indicates index of unique genes. Genes on the top left appeared in all 500 cross-validations, whereas genes at the bottom right appeared only once. Genes appearing in at least 250 networks are marked on the plot.

© 2022 The Authors

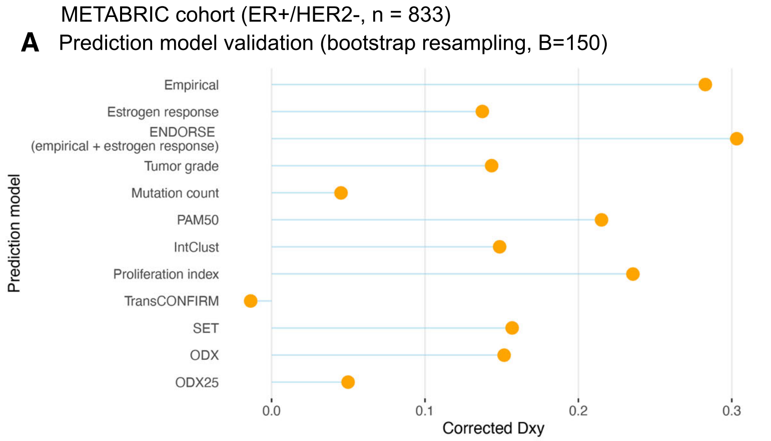

B Model comparisons

Model 1	Model 2	Variance test *H1: Model 1 and Model 2 are distinguishable*	Likelihood ratio test (nested) *H1A: Full model fits better than reduced model*	Partial likelihood ratio test (non-nested) *H1A: Model 1 fits better than Model 2*
ENDORSE	Empirical	1.47×10^{-3}	1.09×10^{-3}	
	Estrogen response	3.83×10^{-6}	3.93×10^{-14}	
	METABRIC tumor characteristics and signatures			
	Tumor grade	$< 2 \times 10^{-16}$		2.08 x 10-3
	Mutation count	$< 2 \times 10^{-16}$		9.76 x 10-6
	PAM50	2.22 x 10-8		0.033
	IntClust	$< 2 \times 10^{-16}$		0.02
	Proliferation index	3.73×10^{-5}		4.42×10^{-5}
	External signatures			
	TransCONFIRM	1.67×10^{-7}		9.73×10^{-6}
	SET	$< 2 \times 10^{-16}$		3.53×10^{-5}
	ODX	2.18×10^{-7}		6.15×10^{-5}
	ODX25	1.49×10^{-6}		1.32×10^{-5}

Figure EV2. Model evaluation and comparison with other predictors in METABRIC.

A Lollipop plots displaying corrected Somer's D_{xy} indices of ENDORSE and various other univariate Cox proportional hazards models. The indices were calculated using 150-fold bootstrap resampling of the training dataset.

B Table comparing the ENDORSE model with various other univariate Cox models using partial likelihood ratio tests. The comparison between the nested ENDORSE model and its two components was performed using a likelihood ratio test, while other non-nested univariate models were compared using a partial likelihood ratio test.

© 2022 The Authors

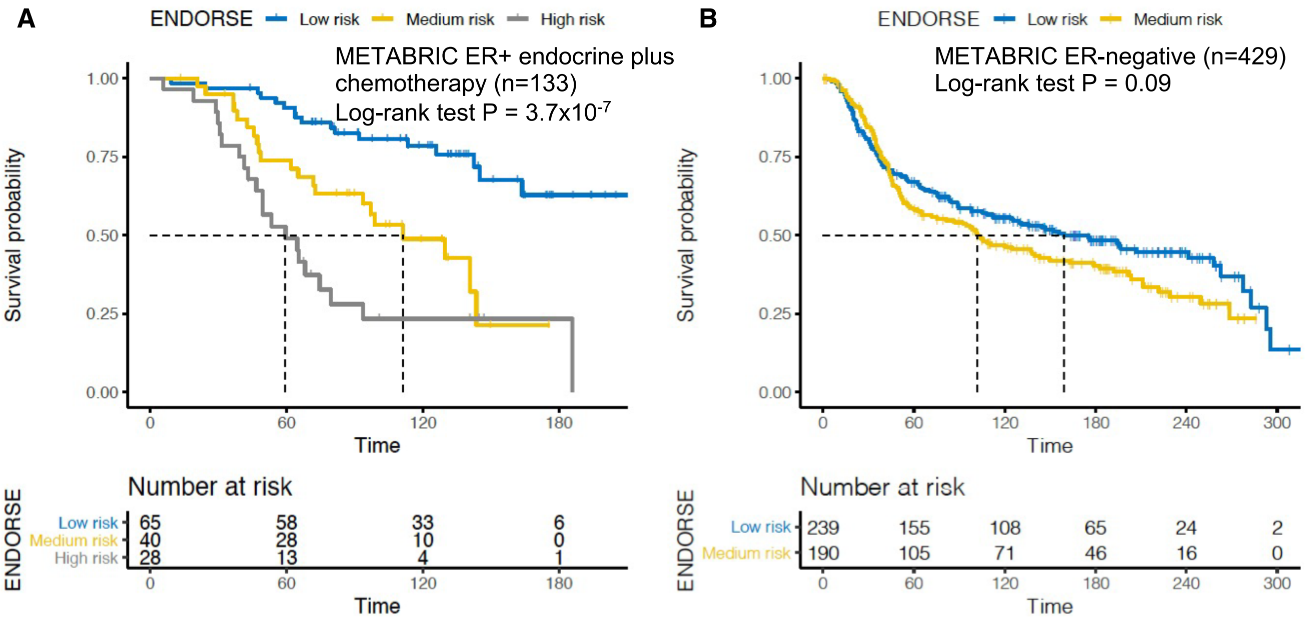

Figure EV3. ENDORSE evaluation in ER$^+$ tumors receiving chemotherapy and ER- tumors.

A, B Kaplan–Meir recurrence-free survival curves and risk tables of METARBIC cohort ER$^+$ patients that received endocrine therapy in (A) combination with chemotherapy (n = 133) and (B) METABRIC ER$^-$ patients (n = 429). In each analysis, the patients were stratified according to ENDORSE predicted risk scores. *P*-values were obtained using log-rank tests.

© 2022 The Authors

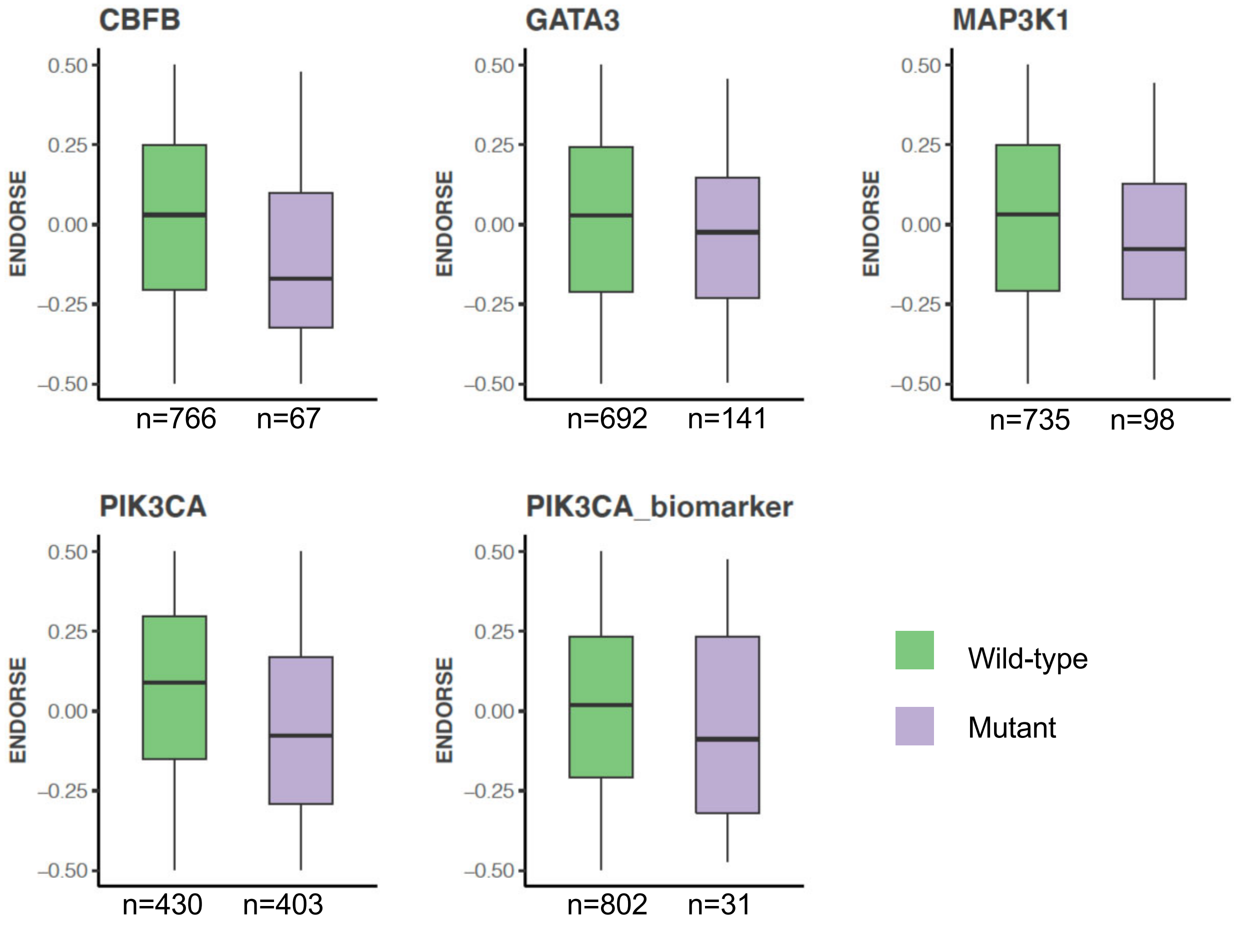

Figure EV4. Somatic variants associated with ENDORSE scores.

Boxplots showing ENDORSE predicted risk scores in METABRIC that were significantly different in mean values (false discovery rate-adjusted $P < 0.05$) in samples grouped by mutation status of cancer genes. The colored boxes display interquartile range with median, while the whiskers show 1.5 × interquartile range, with each sample representing a biological replicate. The numbers of wild type and mutated tumors serving as biological replicates are annotated below the boxplots for each gene.

 © 2022 The Authors

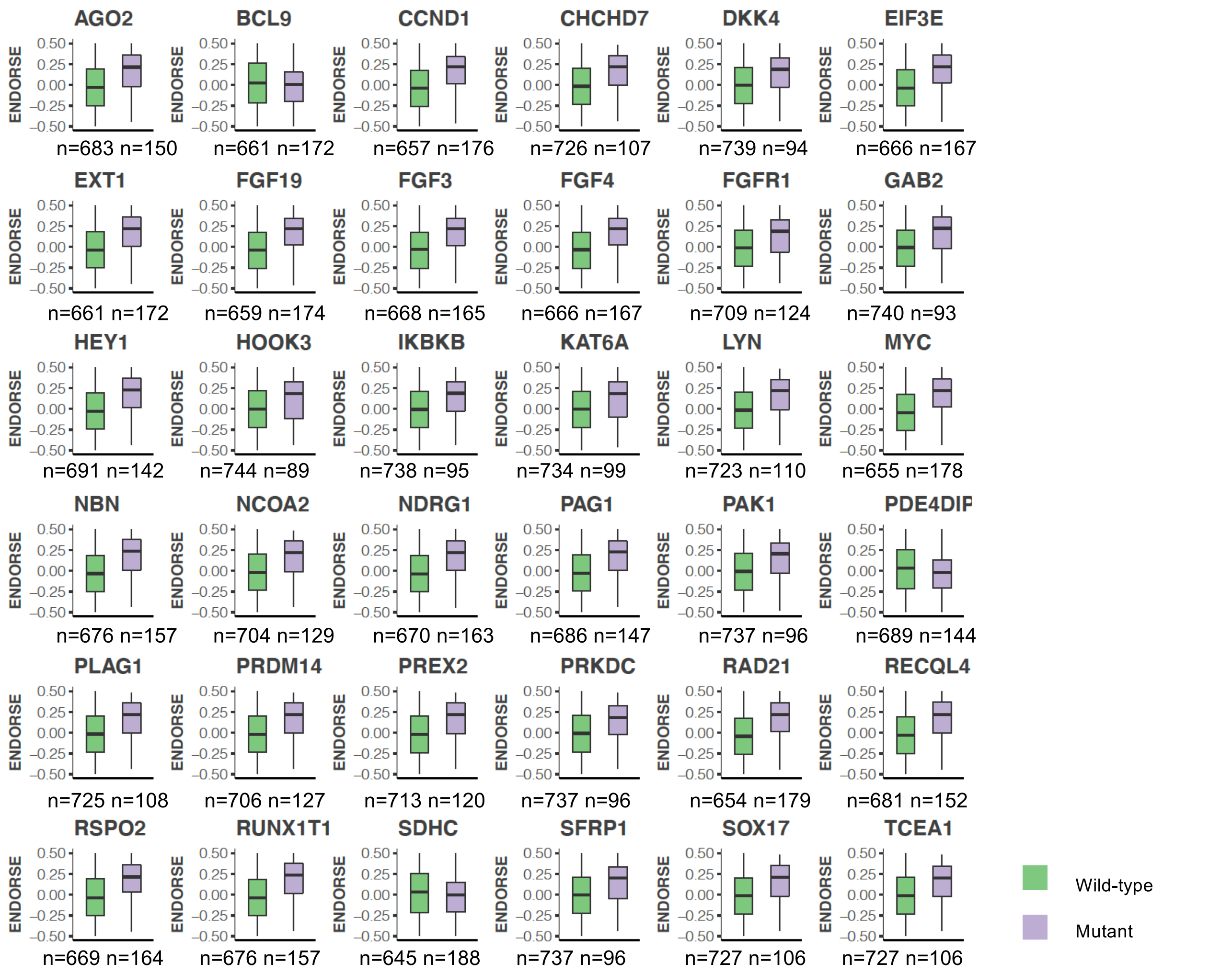

Figure EV5. Copy number amplifications associated with ENDORSE scores.

Boxplots showing ENDORSE predicted risk scores in METABRIC that were significantly different in mean values (false discovery rate-adjusted $P < 0.05$) in samples grouped by copy number amplification status of cancer genes. The colored boxes display interquartile range with median, while the whiskers show 1.5 × interquartile range. The numbers of wild type (no gain) and mutated (copy number amplified) tumors serving as independent biological replicates are annotated below the boxplots for each gene.

© 2022 The Authors
