# Supplementary material for: ENDORSE: a prognostic model for endocrine therapy in estrogen‐receptor‐positive breast cancers
Source: Mol Syst Biol. 2022 Jun 7;18(6):e10558. doi: 10.15252/msb.202110558 (PMC9172932; doi:10.15252/msb.202110558)
Supplement: Supplementary file 17 [file MSB-18-e10558-s005.pdf]

molecular systems biology

ENDORSE: a prognostic model for endocrine therapy in estrogen-receptor positive breast cancers

Aritro Nath, Adam Cohen, and Andrea Bild
DOI: 10.15252/msb.202110558

Corresponding author(s): Andrea Bild (abild@coh.org) , Aritro Nath (aritronath@gmail.com)

Review Timeline:

Submission Date:	17th Nov 21
Editorial Decision:	11th Jan 22
Revision Received:	18th Mar 22
Editorial Decision:	5th May 22
Revision Received:	19th May 22
Accepted:	20th May 22

Editor: Maria Polychronidou

Transaction Report:

(Note: With the exception of the correction of typographical or spelling errors that could be a source of ambiguity, letters and reports are not edited. Depending on transfer agreements, referee reports obtained elsewhere may or may not be included in this compilation. Referee reports are anonymous unless the Referee chooses to sign their reports.)

Thank you again for submitting your work to Molecular Systems Biology. We have now heard back from the three referees who agreed to evaluate your study. As you will see below, the reviewers raise substantial concerns, which prevent the publication of the study in its current form.

Overall, the reviewers acknowledge that the presented prognostic model seems potentially useful. However, they point out that as it stands its performance and value for clinical applications is not well supported. Given that the reviewers acknowledge that the topic of the study is in principle relevant and they all provide constructive and detailed suggestions on how to revise and extend the study, we have decided to offer you a chance to address the issues raised in a major revision.

Without repeating all the points listed below, the most fundamental issues are the following:

- As all three reviewers point out, the prognostic value and superiority of the presented signature/model compared to other signatures and clinical factors need to be better supported.

- Reviewer #2 mentions that validation with an independent dataset would better support the conclusions of the study.

All issues raised by the reviewers need to be satisfactorily addressed. Please contact me in case you would like to discuss in further detail any of the issues raised.

On a more editorial level, we would ask you to address the following points:

REFEREE REPORTS

Reviewer #1:

Summary: Nath A et al.
The authors present a new prognostic model (ENDORSE) for endocrine therapy in advanced ER+ breast cancer. Their model is based on two components a gene expression signature associated with long-term survival and a curated set of genes expressed in response to estrogen exposure. They validate their model and contrast its performance comparing to other established risk prediction models. Furthermore, they try to interpret the different in high and low risk patients predicted by their new score ENDORSE. They used the METABRIC data set focusing on ER+, HER2- patients receiving endocrine treatment without additional chemotherapy (CT) to train their algorithm and selected three validation data sets. As there is a lack of risk prediction models focusing on advanced ER+ breast cancers a new risk prediction model is needed to risk stratify these patients. General remarks
The work presented is providing a technical advancement as ENDORSE is a new risk prediction model focusing on high-risk ER + breast cancer patients. However, the performance of the model should be presented in more detail comparing to established risk predication models. Currently it is difficult to judge the performance of ENDORSE. Clinically a rigorously validated signature will be of interest to improve clinical treatment decisions.
Major points

1. In the performance evaluation of ENDORSE in METABRIC is compared against other signatures and clinical factors. However, the authors trained on the same patients which can be misleading. This should be clearly emphasized as the performance on the training set is typically higher than on validation sets. Therefore, the outperformance of ENDORSE in this setting should be expected and the authors could focus more on the validation data sets.
2. Could the risk in patients receiving CT also be assessed? Which limitations does this patient selection have assuming that higher risk patients in METABRIC have received a combination treatment endocrine and chemotherapy. Furthermore a risk prediction for a broader cohort would be interesting e.g. the full METABRIC-Cohort.
3. The authors focus on a subset of patients and build their model. Is the performance of their model improved due to the selected training set or due to their modelling approach?
4. They select ENDORSE scores of 1 and 2 to stratify patients into risk groups. How were these cutpoints identified are they the most optimal ones?
5. The TransCONFIRM trial is due to the missing survial information not ideal for assessing the ENDORSE performance.
6. The authors did not assess the performance of all clinical risk factors and prognostic signatures on the validation data sets. Including this would enable to assess the performance of ENDORSE in comparison to already established models.
7. Figure 3B how would the risk prediction into two groups compare to the Transconfirm performance. How were the cutoffs to create three risk groups identified?

Minor points
1. Please define the list of "known-cancer related" genes used in assessing the biological features of the ENDORSE score.
2. Please always add the significance for the individual ENDORSE risk groups and not only an overall p-value.

Reviewer #2:

Nath and colleagues have developed a gene expression-based overall survival prognostic model for endocrine-therapy-treated advanced ER+ breast cancers using 833 cases from the METABRIC study (primarily stage 0-2). Their ENDORSE model was shown to have superior performance to other gene expression signatures and clinical covariates when evaluated on the training set. In the TransCONFIRM independent dataset of 112 ER+ stage 4 patients, the ENDORSE model was a significant predictor of percentage of Ki67 staining, a proliferation biomarker, and performed better for Ki67 prediction than the SET or TransCONFIRM models. In the SET independent dataset of 140 ER+ stage 4 patients, the ENDORSE model was a significant predictor of OS and PFS and performed better than the SET model (primarily in identifying the very poor outcome subgroup) and the TransCONFIRM model (which performed poorly). It is quite interesting that ENDORSE identifies an extremely poor prognosis subgroup within the SET cohort (Figure 4). In the ACOSOG independent dataset of 109 ER+ stage 2/3 patients, the ENDORSE model was a significant predictor of baseline and end-of-treatment Ki67 percentage, associated significantly to

clinical response, and outperformed the SET and TransCONFIRM models. Finally, the ENDORSE model was investigated for associations to biological gene expression signatures, SNVs, and CNAs, and a number of significant pathways, genes, and loci were identified. The manuscript is generally clear and well written. The data presented appears generally sound and generally supports their conclusions, although there are some significant areas which should be addressed:
1. A primary weakness is that there is no proper external independent validation for overall survival in a patient population that is similar to the training set. The external datasets used are either stage 4 (and thus are a very different clinical situation), or do not have the relevant OS variable. Moreover, the Ki67 variable is used as an outcome proxy, and for the purpose of evaluating an OS prognostic model, Ki67 is insufficient to truly say the model has been validated. The authors should try and identify a more relevant independent dataset (or generate their own) which has the OS outcome variable.
2. In ER+ breast cancer, the biological determinants of early relapse and late relapse may be different. For example, early relapse may be due to inherent resistance and/or high proliferation and early dissemination, whereas late relapse may be due to reactivation of dormant clones and immune evasion. This should be addressed or commented on in some way.
3. The type of input data, platforms, and issues translating or adapting the models between platforms and datasets should be discussed in more detail.
4. Related to the above, the faithfulness of the authors' recreation of the ODX and ODX25 signatures to the actual performance what OncoTypeDX would be on the evaluated tumors is unclear. Do you have any estimate of how well these two surrogates perform compared to the real test?
5. It would be helpful that the authors discuss the choice of using Somers' D statistic rather than any other more commonly used metric.

Reviewer #3:

Using the Metabric ER+ breast cancer microarray dataset, the authors present an endocrine therapy prognostic model for ER+ breast cancers, potentially also relevant for advanced breast cancers. The model (ENDORSE) is composed of two components: an empirical signature derived from overall survival analyses in the depicted dataset, and an estrogen response signature.

Therefore, it is expected that the model captures more information that just ER-associated genes, so comparing it to a single factor (eg Ki67) would not be appropriate. Also, as it was developed in Metabric, the model may not be specific to advanced disease.

The model is able to successfully predict outcomes and associations with proliferation (ki67) in two independent studies of advanced breast cancer (TransCONFIRM and SET(ER/PR). However, the comparison between ENDORSE and the TransCONFIRM score generated by the authors may appear unfair: the outcome measures were different, and the therapy, despite being fulvestrant (TransCONFIRM), is not uniform in both datasets (Metabric vs TransCONFIRM). Most important, tumors TransCONFIRM were resistant to previous antiestrogen therapy, so it was clearly not biologically comparable to Metabric tumors. In turn, ENDORSE nicely captures Ki67 status in TransCONFIRM, but this observation simply reflects that ENDORSE embraces proliferation information, which is required for prediction, as the authors acknowledge earlier in published signatures.

The comparison with the SET(ER/PR) study may also appear unfair. SET(ER/PR) was focused on ESR1-PGR correlated genes, so it is also expected to capture a partially different "phenotype" to ENDORSE, which includes an estrogen related signature, but adds other information (empirical). Nonetheless, the initial aim of SET(ER/PR) was to provide a relatively simple and applicable gene expression platform for clinical use.
Then, while ENDORSE slightly outperforms PAM50, which is the magnitude of this difference of predictive power?

As acknowledged by authors, and consistent with the different design of the TransCONFIRM ad SET(ER/PR) studies, the signatures of these publications were not interchangeable, but ENDORSE did work fairly well in both. Again, this indicates that ENDORSE is powerful to predict outcome of ER+ breast cancer, although it is not specifically designed/focused on advanced disease.

As a third dataset, the authors use the clinical trial data from neoadjuvant treatment with different aromatase inhibitors in cases with stage II_III disease, but not IV (metastatic besides lymph nodes) stage. ENDORSE works well to classify proliferation and response in this set, which is consistent again with the original design, but not specific of advanced disease.

Finally, the pathways associated with ENDORSE are fully coherent with established knowledge of signatures that are associated with response to endocrine therapy in primary and advanced disease: mTOR, MYC, proliferation E2F/RB1.

Overall, the study partially mixes study designs and disease stages to demonstrate the usefulness of another signature in ER+ breast cancer outcome. However, it is unclear the specific relevance in advanced disease with uniform features, therapy and outcome evlaution.

Reviewer #1:

Summary: Nath A et al.

The authors present a new prognostic model (ENDORSE) for endocrine therapy in advanced ER+ breast cancer. Their model is based on two components a gene expression signature associated with long-term survival and a curated set of genes expressed in response to estrogen exposure. They validate their model and contrast its performance comparing to other established risk prediction models. Furthermore, they try to interpret the different in high and low risk patients predicted by their new score ENDORSE. They used the METABRIC data set focusing on ER+, HER2- patients receiving endocrine treatment without additional chemotherapy (CT) to train their algorithm and selected three validation data sets. As there is a lack of risk prediction models focusing on advanced ER+ breast cancers a new risk prediction model is needed to risk stratify these patients.

General remarks

The work presented is providing a technical advancement as ENDORSE is a new risk prediction model focusing on high-risk ER+ breast cancer patients. However, the performance of the model should be presented in more detail comparing to established risk predication models. Currently it is difficult to judge the performance of ENDORSE. Clinically a rigorously validated signature will be of interest to improve clinical treatment decisions.

Major points

1. In the performance evaluation of ENDORSE in METABRIC is compared against other signatures and clinical factors. However, the authors trained on the same patients which can be misleading. This should be clearly emphasized as the performance on the training set is

typically higher than on validation sets. Therefore, the outperformance of ENDORSE in this setting should be expected and the authors could focus more on the validation data sets.

We acknowledge the pertinent point raised by the reviewer and have updated the performance evaluation in METABRIC to mitigate these concerns. As the METABRIC dataset provided the most comprehensive genomic and clinical annotation, most of which were not available for other public datasets. Owing to this limitation, we focused on improving the validation framework on the METABRIC data. To demonstrate the performance of the ENDORSE model in METABRIC, we first split the cohort into a training (n=416) and a hold-out test/validation (n=417) subset. The training and test subsets had no significant differences in key clinical features, like stage, grade, and mutation burden.

We next obtained the empirical signature using a LASSO-regularized Cox proportional hazards model, as originally described, but now only using the training subset. Subsequently, we calculated the gene set enrichment scores of the empirical signature and the estrogen response signature, followed by fitting a Cox model using only the training subset. The coefficients from this model were used to predict the ENDORSE scores in the hold-out validation subset. Then, we compared the performance of ENDORSE in the validation subset with other clinical predictors and existing signatures using bootstrap resampling, as previously described in the manuscript. Using this new framework, we validated that the ENDORSE model performed better than clinical factors and prognostic signatures.

This new framework is shown in the new Figure 2A and described in the Methods section on Pages 17-18, lines 419-432. The associated results are described on Pages 5-7, lines 110-154.

Finally, we applied the ENDORSE model to predict 10-year recurrence free survival using baseline tumor transcriptome from an additional clinical cohort. The Oxford cohort consisted of similar key pathological features where 31% tumors were classified as grade 3 as compared to 35% in METABRIC. Performance evaluation of ENDORSE in this dataset revealed significant difference in the survival curves of the ENDORSE risk groups ($P = 2.4 \times 10^{-9}$) while both the SET (P = 0.5) and TransCONFIRM (P = 0.3) signatures failed. These new results are shown in the new Figure 3 and described on Page 8, lines 174-182.

2. Could the risk in patients receiving CT also be assessed? Which limitations does this patient selection have assuming that higher risk patients in METABRIC have received a combination treatment endocrine and chemotherapy. Furthermore a risk prediction for a broader cohort would be interesting e.g. the full METABRIC-Cohort.

As suggested by the reviewer, we analyzed the ENDORSE risk scores for patients who have received chemotherapy and endocrine therapy in the METABRIC study (n=133). The ENDORSE score resulted in significant difference in the survival curves of the strata ($P=3.7 \times 10^{-7}$). This new information has been displayed in Figure EV3 and described in results section on Page 7, lines 160-162.

We agree with the reviewer that risk prediction the broader METABRIC cohort are important and interesting questions; however, these have been previously addressed in published studies (eg: https://www.ncbi.nlm.nih.gov/pubmed/23395906, https://www.ncbi.nlm.nih.gov/pubmed/29100405, https://doi.org/10.3390/cells9071643). Given this information and unmet clinical needs, our current study objectives were limited to building a prognostic model for ER+ patients receiving endocrine therapy across all stages and grades.

3. The authors focus on a subset of patients and build their model. Is the performance of their model improved due to the selected training set or due to their modelling approach?

As our study objective was to build a prognostic model for all ER+ breast cancers on endocrine therapy, including advanced and metastatic tumors, we focused on building the model using the most relevant subset of METABRIC patients as displayed in Figure 1A. Along with the empirical signature that was derived using this subset of patients, the ENDORSE model also included a curated estrogen response signature. Thus, from a biological perspective, it was appropriate to train the model on ER+ patients. In our independent validation analyses, we showed that ENDORSE vastly outperformed other prognostic models. Thus, the superior performance is attributed to both the selection of an appropriate cohort of patients to train the model and the modeling approach.

4. They select ENDORSE scores of 1 and 2 to stratify patients into risk groups. How were these cutpoints identified are they the most optimal ones?

We used ENDORSE scores of 1 and 2 to as examples, as stated in the first section of the results on Page 5, lines 105-108. The motivation behind using the stratified risk groups was to provide an example of a potential guideline for clinicians to decide whether a patient will benefit from endocrine therapy. In our examples, an ENDORSE score of 2 indicates that a particular patient is twice as likely to die on endocrine therapy compared to the baseline. With this information, a clinician could decide whether the patient should receive endocrine therapy alone, in combination with targeted therapy against estrogen resistance pathways or switch to chemotherapy.

For clinical application, validation in a clinical trial will be necessary to determine optimal cutoffs, where patients are stratified into treatment arms based on ENDORSE scores. To this end, we have opened a clinical trial called SPOCK (Systems Biology Guided Therapy for Breast Cancer Positive for Oestrogen Receptor After Aromatase Inhibitor and CDK Inhibition). This Phase II trial for progressive metastatic ER+ breast cancers will utilize the ENDORSE model to identify which patients are most likely to benefit from continued endocrine therapy vs. switching to chemotherapy. (https://www.clinicaltrials.gov/ct2/show/NCT04965688)

5. The TransCONFIRM trial is due to the missing survial information not ideal for assessing the ENDORSE performance.

While we agree with the reviewer that the TransCONFIRM trial is not ideal, it still provided us with an opportunity to compare ENDORSE with the TransCONFIRM model, on the same dataset that the TransCONFIRM model was originally developed with. As the reviewer stated in Point 1, it is not surprising that a model trained on the same training dataset outperforms independent signatures, this provided us with an opportunity to compare the ENDORSE model to this published signature. Results from this analysis show that ENDORSE outperforms the TransCONFIRM model, on the same dataset that TransCONFIRM was developed with (Figure 4, Page 8, lines 184-196).

6. The authors did not assess the performance of all clinical risk factors and prognostic signatures on the validation data sets. Including this would enable to assess the performance of ENDORSE in comparison to already established models.

We agree with this reviewer’s point. Unfortunately, very limited clinical information is available for the independent validation datasets. Due to this limitation, it was not possible to comprehensively assess all clinical risk factors in the external validation datasets. However, we have addressed this issue in our new validation framework in the METABRIC analysis (Figure 2A) by training the model independently in a training subset and only comparing the performance of the model in the hold-out test subset against other clinical risk factors and published signatures.

7. Figure 3B how would the risk prediction into two groups compare to the Transconfirm performance. How were the cutoffs to create three risk groups identified?

We used the mean ENDORSE scores of the cohort to split them into two groups. This stratification yielded a significant difference in the groups (P=0.02), which outperformed the TransCONFIRM score (P=0.05). The cutoffs for the three risk groups described in the results were consistent with other analyses (1 for medium risk and 2 for high risk). We have updated the methods section to reflect this information on Page 19, lines 456-458).

Minor points

1. Please define the list of "known-cancer related" genes used in assessing the biological features of the ENDORSE score.

The list of known cancer-related genes was obtained from the Catalogue of Somatic Mutations in Cancer (COSMIC) cancer gene census (Sondka et al, 2018; Tate et al, 2019). This information was added to the methods section on Page 19, lines 477-478.

2. Please always add the significance for the individual ENDORSE risk groups and not only an overall p-value.

We have updated the results section with additional information comparing individual ENDORSE risk groups. For survival analyses including patients stratified into three groups using ENDORSE, we have updated the results section to include the P-values from the Cox proportional hazards analysis. For the categorical analyses, we have updated the results section to include P-values from Tukey HSD posthoc test following ANOVA.

These updates were at the following locations in the results section:

Page 5, line 107

Page 6, line 119

Page 8, line 180

Page 8, line 193

Page 9, line 202

Page 9, line 205

Page 9, lines 216-218

Reviewer #2:

Nath and colleagues have developed a gene expression-based overall survival prognostic model for endocrine-therapy-treated advanced ER+ breast cancers using 833 cases from the METABRIC study (primarily stage 0-2). Their ENDORSE model was shown to have superior performance to other gene expression signatures and clinical covariates when evaluated on the training set. In the TransCONFIRM independent dataset of 112 ER+ stage 4 patients, the ENDORSE model was a significant predictor of percentage of Ki67 staining, a proliferation biomarker, and performed better for Ki67 prediction than the SET or TransCONFIRM models. In the SET independent dataset of 140 ER+ stage 4 patients, the ENDORSE model was a significant predictor of OS and PFS and performed better than the SET model (primarily in identifying the very poor outcome subgroup) and the TransCONFIRM model (which performed poorly). It is quite interesting that ENDORSE identifies an extremely poor prognosis subgroup within the SET cohort (Figure 4). In the ACOSOG independent dataset of 109 ER+ stage 2/3 patients, the ENDORSE model was a significant predictor of baseline and end-of-treatment Ki67 percentage, associated significantly to clinical response, and outperformed the SET and TransCONFIRM models. Finally, the ENDORSE model was investigated for associations to biological gene expression signatures, SNVs, and CNAs, and a number of significant pathways, genes, and loci were identified. The manuscript is generally clear and well written. The data presented appears generally sound and generally supports their conclusions, although there are some significant areas which should be addressed:

1. A primary weakness is that there is no proper external independent validation for overall survival in a patient population that is similar to the training set. The external datasets used are either stage 4 (and thus are a very different clinical situation), or do not have the relevant OS variable. Moreover, the Ki67 variable is used as an outcome proxy, and for the purpose of

evaluating an OS prognostic model, Ki67 is insufficient to truly say the model has been validated. The authors should try and identify a more relevant independent dataset (or generate their own) which has the OS outcome variable.

We acknowledge the pertinent point raised by the reviewer and have updated the performance evaluation in METABRIC to mitigate these concerns. As the METABRIC dataset provided the most comprehensive genomic and clinical annotation, most of which were not available for other public datasets. Owing to this limitation, we focused on improving the validation framework on the METABRIC data. To demonstrate the performance of the ENDORSE model in METABRIC, we first split the cohort into a training (n=416) and a hold-out test/validation (n=417) subset. The training and test subsets had no significant differences in key clinical features, like stage, grade, and mutation burden.

We next obtained the empirical signature using a LASSO-regularized Cox proportional hazards model, as originally described, but now only using the training subset. Subsequently, we calculated the gene set enrichment scores of the empirical signature and the estrogen response signature, followed by fitting a Cox model using only the training subset. The coefficients from this model were used to predict the ENDORSE scores in the hold-out validation subset. Then, we compared the performance of ENDORSE in the validation subset with other clinical predictors and existing signatures using bootstrap resampling, as previously described in the manuscript. Using this new framework, we validated that the ENDORSE model performed better than clinical factors and prognostic signatures.

This new framework is shown in the new Figure 2A and described in the Methods section on Pages 17-18, lines 419-432. The associated results are described on Pages 5-7, lines 110-154.

Finally, we applied the ENDORSE model to predict 10-year recurrence free survival using baseline tumor transcriptome from an additional clinical cohort. The Oxford cohort consisted of similar key pathological features where 31% tumors were classified as grade 3 as compared to 35% in METABRIC. Performance evaluation of ENDORSE in this dataset revealed significant difference in the survival curves of the ENDORSE risk groups ($P = 2.4 \times 10^{-9}$) while both the SET (P = 0.5) and TransCONFIRM (P = 0.3) signatures failed. These new results are shown in the new Figure 3 and described on Page 8, lines 174-182.

2. In ER+ breast cancer, the biological determinants of early relapse and late relapse may be different. For example, early relapse may be due to inherent resistance and/or high proliferation and early dissemination, whereas late relapse may be due to reactivation of dormant clones and immune evasion. This should be addressed or commented on in some way.

The reviewer brings up a pertinent point in that the mechanisms of early and late relapse are likely different. We explored the key pathways associated with an ENDORSE predicted risk >1 and significantly enriched in tumors with early mortality (<5 years overall survival) vs. late mortality (>5 years overall survival). Our analysis revealed that early mortality tumors showed significant enrichment of key pathways associated with endocrine resistance, stemness, angiogenesis and epithelial to mesenchymal transition. On the other hand, late mortality tumors were dependent on estrogen signaling at the time of sample collection and were not enriched in specific resistance pathways, suggesting these tumors acquire various resistance mechanisms over time.

We added the following description in the results section on Page 11:

“Finally, we evaluated whether the ENDORSE model reflects different key phenotypes associated with early (within 5 years) vs. late (beyond 5 years) mortality. For patients with an ENDORSE risk > 1, we found that tumors associated with late mortality showed elevated estrogen response signatures whereas tumors linked with early mortality showed enrichment of epithelial to mesenchymal transition, stemness, angiogenesis and related signaling pathways that are known to contribute to these phenotypes including TGFB, YAP, MEK and AKT pathways (EV Datasets 13-15).”

We added the following discussion on Page 13:

“In the METABRIC cohort, tumors with early mortality (within 5 years of sample collection) showed consistent enrichment of pathways that are known to contribute to epithelial to mesenchymal transition, angiogenesis and stemness. Enriched pathways, including TGF-beta (Band & Laiho, 2011) YAP (Ma et al, 2022) have been suggested to directly cross-talk and repress the estrogen receptor signaling pathway. Concurrently, MEK (Fujii et al, 2011) and PI3K/AKT/MTOR (Ciruelos Gil, 2014) have been implicated as key primary resistance mechanisms in endocrine resistant ER+ breast cancers. In contrast, tumors with late mortality did not show enrichment of specific signaling pathways, suggesting diverse resistance mechanism may evolve long-term and contribute to the late relapse of these tumors. Characterizing the heterogeneity in late relapse tumors would be necessary to understand the diverse mechanisms that lead to evolution of resistance in these tumors (Zardavas et al, 2015).”

3. The type of input data, platforms, and issues translating or adapting the models between platforms and datasets should be discussed in more detail.

Thank you for highlighting this important point. We found that three key elements are important for successfully translating the model across datasets profiled using different platforms. First, we

pre-processed the gene expression datasets such that they roughly followed a similar distribution (i.e., log transformed and scaled). Second, we performed batch-correction to account for as much remaining variance as possible that could be attributed to differences in batches. Third, we used a rank-based gene set enrichment approach, which calculates enrichment scores using the empirical cumulative distribution functions of the ranks of the genes in a signature. This also helps in mitigating the differences in expression distributions across platforms. We have updated the methods to include this information in the relevant sections. (Page 15, lines 355-360; Page 16-17, lines 396-401; Page 18, lines 440-442).

4. Related to the above, the faithfulness of the authors' recreation of the ODX and ODX25 signatures to the actual performance what OncoTypeDX would be on the evaluated tumors is unclear. Do you have any estimate of how well these two surrogates perform compared to the real test?

We have used the formula provided by the original publication describing the signature to recreate the signatures. Unfortunately, in the absence of public transcriptomic data with actual OncoTypeDx test results available from the same samples, we cannot comment how the surrogates compare with the test results. However, the TransCONFIRM study also evaluated the OncoTypeDX test scores in comparison with their signature. The study found that the test performed poorly (PFS P=0.31 and OS P=0.58) in advanced ER+ patients receiving fulvestrant. (https://www.ncbi.nlm.nih.gov/pubmed/27185372). Although indirect, this also suggests that ENDORSE is a better model than OncoTypeDx for the advanced tumors, as it clearly outperforms the TransCONFIRM model.

5. It would be helpful that the authors discuss the choice of using Somers' D statistic rather than any other more commonly used metric.

We used Somer's D as it provides both a measure of effect size and performance indicator for the agreement between predicted risk (a continuous variable) with survival data of patients (a binary variable). Other commonly used metrics for similar effect size measurements, such as C-index (concordance index) and Kendall's tau can be both calculated directly from Somer's D (see: https://doi.org/10.1177/1536867X0600600302)

Reviewer #3:

Using the Metabric ER+ breast cancer microarray dataset, the authors present an endocrine therapy prognostic model for ER+ breast cancers, potentially also relevant for advanced breast cancers. The model (ENDORSE) is composed of two components: an empirical signature derived from overall survival analyses in the depicted dataset, and an estrogen response signature. Therefore, it is expected that the model captures more information that just ER-associated genes, so comparing it to a single factor (eg Ki67) would not be appropriate. Also, as it was developed in Metabric, the model may not be specific to advanced disease.

We appreciate the reviewer's concern and have updated our objective statement to clearly state that our objective was to develop a prognostic model for all ER+ cancers on endocrine therapy, including advanced and metastatic tumors, regardless of tumor stage, grade, or node status. (See Page 5, lines 88-89). As the current prognostic tests used in the clinic are limited to early-stage, node-negative disease, the ENDORSE model aims to fulfil an unmet clinical need for a biomarker that can used for advanced and metastatic disease in addition to early-stage disease.

The model is able to successfully predict outcomes and associations with proliferation (ki67) in two independent studies of advanced breast cancer (TransCONFIRM and SET(ER/PR). However, the comparison between ENDORSE and the TransCONFIRM score generated by the authors may appear unfair: the outcome measures were different, and the therapy, despite being fulvestrant (TransCONFIRM), is not uniform in both datasets (Metabric vs TransCONFIRM). Most important, tumors TransCONFIRM were resistant to previous antiestrogen therapy, so it was clearly not biologically comparable to Metabric tumors. In turn, ENDORSE nicely captures Ki67 status in TransCONFIRM, but this observation simply reflects

that ENDORSE embraces proliferation information, which is required for prediction, as the authors acknowledge earlier in published signatures.

The comparison with the SET(ER/PR) study may also appear unfair. SET(ER/PR) was focused on ESR1-PGR correlated genes, so it is also expected to capture a partially different "phenotype" to ENDORSE, which includes an estrogen related signature, but adds other information (empirical). Nonetheless, the initial aim of SET(ER/PR) was to provide a relatively simple and applicable gene expression platform for clinical use.

We agree that the validation datasets were not directly comparable to the METABRIC training dataset. While the TransCONFIRM and SET ER/PR applications demonstrate the superior performance of ENDORSE in external validation datasets with advanced stage and metastatic disease, we have performed additional analyses to validate the performance of METABRIC in a biologically similar setting. We have updated the performance evaluation framework in METABRIC, by first splitting the cohort into a training (n=416) and a hold-out test/validation (n=417) subset. The training and test subsets had no significant differences in key clinical features, like stage, grade, and mutation burden.

We next obtained the empirical signature using a LASSO-regularized Cox proportional hazards model, as originally described, but now only using the training subset. Subsequently, we calculated the gene set enrichment scores of the empirical signature and the estrogen response signature, followed by fitting a Cox model using only the training subset. The coefficients from this model were used to predict the ENDORSE scores in the hold-out validation subset. Then, we compared the performance of ENDORSE in the validation subset with other clinical predictors and existing signatures using bootstrap resampling, as previously described in the

manuscript. Using this new framework, we validated that the ENDORSE model performed better than clinical factors and prognostic signatures.

This new framework is shown in the new Figure 2A and described in the Methods section on Pages 17-18, lines 419-432. The associated results are described on Pages 5-7, lines 110-154.

Finally, we applied the ENDORSE model to predict 10-year recurrence free survival using baseline tumor transcriptome from an additional clinical cohort. The Oxford cohort consisted of similar key pathological features where 31% tumors were classified as grade 3 as compared to 35% in METABRIC. Performance evaluation of ENDORSE in this dataset revealed significant difference in the survival curves of the ENDORSE risk groups ($P = 2.4 \times 10^{-9}$) while both the SET ($P = 0.5$) and TransCONFIRM ($P = 0.3$) signatures failed. These new results are shown in the new Figure 3 and described on Page 8, lines 174-182.

Then, while ENDORSE slightly outperforms PAM50, which is the magnitude of this difference of predictive power?

The performance of ENDORSE ($D_{xy} = 0.351$) in validation subset was nearly 48% greater than PAM50 ($D_{xy} = 0.237$).

As acknowledged by authors, and consistent with the different design of the TransCONFIRM ad SET(ER/PR) studies, the signatures of these publications were not interchangeable, but ENDORSE did work fairly well in both. Again, this indicates that ENDORSE is powerful to predict outcome of ER+ breast cancer, although it is not specifically designed/focused on advanced disease.

As a third dataset, the authors use the clinical trial data from neoadjuvant treatment with different aromatase inhibitors in cases with stage II_III disease, but not IV (metastatic besides lymph nodes) stage. ENDORSE works well to classify proliferation and response in this set, which is consistent again with the original design, but not specific of advanced disease.

Finally, the pathways associated with ENDORSE are fully coherent with established knowledge of signatures that are associated with response to endocrine therapy in primary and advanced disease: mTOR, MYC, proliferation E2F/RB1.

Overall, the study partially mixes study designs and disease stages to demonstrate the usefulness of another signature in ER+ breast cancer outcome. However, it is unclear the specific relevance in advanced disease with uniform features, therapy and outcome evaluation.

As described above, we have performed additional analyses to validate the performance of ENDORSE in biological similar disease state as the training cohort and have provided evidence that the model is better than existing models for advanced disease as well. These results provide evidence that the ENDORSE model fulfils our original objective of developing a prognostic model for all ER+ cancers on endocrine therapy, including advanced and metastatic tumors, regardless of tumor stage, grade, or node status.

Furthermore, we have opened a clinical trial called SPOCK (Systems Biology Guided Therapy for Breast Cancer Positive for Oestrogen Receptor After Aromatase Inhibitor and CDK Inhibition). This Phase II trial for progressive metastatic ER+ breast cancers will utilize the ENDORSE model to identify which patients are most likely to benefit from continued endocrine therapy alone or in combination with targeted inhibitors against PI3K or MTOR signaling

pathways vs. switching to chemotherapy. (https://www.clinicaltrials.gov/ct2/show/NCT04965688).

Thank you for sending us your revised manuscript. We have now heard back from the three reviewers who agreed to evaluate your revised study. As you will see below, the reviewers think that the study has improved as a result of the performed revisions and they are overall supportive. However, reviewer #1 lists a few remaining concerns, which we would ask you to address in a final round of revision.

Moreover, we would ask you to address some editorial issues listed below.

REFEREE REPORTS

Reviewer #1:

The authors have substantially improved the manuscript by splitting the METABRIC cohort into test and training data. In addition, they now include an analysis of the patients receiving chemotherapy and endocrine therapy showing a significant difference in survival.

Major points

• p.7 line 152-155

Figure EV2 A is presented to show the improved performance of ENDORSE is based on an analysis were samples used for training are included in accessing the performance which should be avoided or clearly stated. Could the analysis be performed on the validation set only?

Minor points

• In the author checklist the section where the information on the REMARK reporting guidelines is provided could be included.

Reviewer #2:

The authors have addressed this reviewer's concerns adequately and I have no additional comments.

Reviewer #3:

The authors have improved the analyses and presentation. In particular with execution of a complementary analytical approach in Metabric, and the use of the Oxford dataset. Despite limitation of similar datasets as Metabric, the superior performance of ENDORSE is demonstrated.

The authors have made all requested editorial changes.

Accepted **20th May 2022**

Thank you again for sending us your revised manuscript. We are now satisfied with the modifications made and I am pleased to inform you that your paper has been accepted for publication.

EMBO Press Author Checklist

Corresponding Author Name: Andrea Bild	
Journal Submitted to: Molecular Systems Biology	
Manuscript Number: MSB-2021-10558RR	

USEFUL LINKS FOR COMPLETING THIS FORM
The EMBO Journal - Author Guidelines
EMBO Reports - Author Guidelines
Molecular Systems Biology - Author Guidelines
EMBO Molecular Medicine - Author Guidelines

Reporting Checklist for Life Science Articles (updated January 2022)
This checklist is adapted from Materials Design Analysis Reporting (MDAR) Checklist for Authors. MDAR establishes a minimum set of requirements in transparent reporting in the life sciences (see Statement of Task: 10.31222/osf.io/9sm4x). Please follow the journal's guidelines in preparing your manuscript.
Please note that a copy of this checklist will be published alongside your article.

Abridged guidelines for figures
1. Data
The data shown in figures should satisfy the following conditions:
- the data were obtained and processed according to the field's best practice and are presented to reflect the results of the experiments in an accurate and unbiased manner.
- ideally, figure panels should include only measurements that are directly comparable to each other and obtained with the same assay.
- plots include clearly labeled error bars for independent experiments and sample sizes. Unless justified, error bars should not be shown for technical replicates.
- if n<5, the individual data points from each experiment should be plotted. Any statistical test employed should be justified.
- Source Data should be included to report the data underlying figures according to the guidelines set out in the authorship guidelines on Data Presentation.

2. Captions
Each figure caption should contain the following information, for each panel where they are relevant:
- a specification of the experimental system investigated (eg cell line, species name).
- the assay(s) and method(s) used to carry out the reported observations and measurements.
- an explicit mention of the biological and chemical entity(ies) that are being measured.
- an explicit mention of the biological and chemical entity(ies) that are altered/varied/perturbed in a controlled manner.
- the exact sample size (n) for each experimental group/condition, given as a number, not a range;
- a description of the sample collection allowing the reader to understand whether the samples represent technical or biological replicates (including how many animals, litters, cultures, etc.).
- a statement of how many times the experiment shown was independently replicated in the laboratory.
- definitions of statistical methods and measures:
  - common tests, such as t-test (please specify whether paired vs. unpaired), simple χ2 tests, Wilcoxon and Mann-Whitney tests, can be unambiguously identified by name only, but more complex techniques should be described in the methods section;
  - are tests one-sided or two-sided?
  - are there adjustments for multiple comparisons?
  - exact statistical test results, e.g., P values = x but not P values < x;
  - definition of 'center values' as median or average;
  - definition of error bars as s.d. or s.e.m.

Please complete ALL of the questions below.
Select "Not Applicable" only when the requested information is not relevant for your study.

Materials

Newly Created Materials	Information included in the manuscript?	In which section is the information available? (Reagents and Tools Table, Materials and Methods, Figures, Data Availability Section)
New materials and reagents need to be available; do any restrictions apply?	Not Applicable	

Antibodies	Information included in the manuscript?	In which section is the information available? (Reagents and Tools Table, Materials and Methods, Figures, Data Availability Section)
For **antibodies** provide the following information:<br>- Commercial antibodies: RRID (if possible) or supplier name, catalogue number and or/clone number<br>- Non-commercial: RRID or citation	Not Applicable	

DNA and RNA sequences	Information included in the manuscript?	In which section is the information available? (Reagents and Tools Table, Materials and Methods, Figures, Data Availability Section)
**Short novel DNA or RNA including primers, probes**: provide the sequences.	Not Applicable	

Cell materials	Information included in the manuscript?	In which section is the information available? (Reagents and Tools Table, Materials and Methods, Figures, Data Availability Section)
**Cell lines:** Provide species information, strain. Provide accession number in repository **OR** supplier name, catalog number, clone number, and/**OR** RRID.	Not Applicable	
**Primary cultures:** Provide species, strain, sex of origin, genetic modification status.	Not Applicable	
Report if the cell lines were recently **authenticated** (e.g., by STR profiling) and tested for mycoplasma contamination.	Not Applicable	

Experimental animals	Information included in the manuscript?	In which section is the information available? (Reagents and Tools Table, Materials and Methods, Figures, Data Availability Section)
**Laboratory animals or Model organisms:** Provide species, strain, sex, age, genetic modification status. Provide accession number in repository **OR** supplier name, catalog number, clone number, **OR** RRID.	Not Applicable	
**Animal observed in or captured from the field:** Provide species, sex, and age where possible.	Not Applicable	
Please detail **housing and husbandry conditions**.	Not Applicable	

Plants and microbes	Information included in the manuscript?	In which section is the information available? (Reagents and Tools Table, Materials and Methods, Figures, Data Availability Section)
**Plants:** provide species and strain, ecotype and cultivar where relevant, unique accession number if available, and source (including location for collected wild specimens).	Not Applicable	
**Microbes:** provide species and strain, unique accession number if available, and source.	Not Applicable	

Human research participants	Information included in the manuscript?	In which section is the information available? (Reagents and Tools Table, Materials and Methods, Figures, Data Availability Section)
If collected and within the bounds of privacy constraints report on age, sex and gender or ethnicity for all study participants.	Not Applicable	

Core facilities	Information included in the manuscript?	In which section is the information available? (Reagents and Tools Table, Materials and Methods, Figures, Data Availability Section)
If your work benefited from core facilities, was their service mentioned in the acknowledgments section?	Not Applicable	

Design

Study protocol	Information included in the manuscript?	In which section is the information available? (Reagents and Tools Table, Materials and Methods, Figures, Data Availability Section)
If study protocol has been **pre-registered, provide DOI in the manuscript**. For clinical trials, provide the trial registration number **OR** cite DOI.	Not Applicable	
Report the **clinical trial registration number** (at ClinicalTrials.gov or equivalent), where applicable.	Not Applicable	

Laboratory protocol	Information included in the manuscript?	In which section is the information available? (Reagents and Tools Table, Materials and Methods, Figures, Data Availability Section)
Provide DOI OR other citation details if **external detailed step-by-step protocols** are available.	Not Applicable	

Experimental study design and statistics	Information included in the manuscript?	In which section is the information available? (Reagents and Tools Table, Materials and Methods, Figures, Data Availability Section)
Include a statement about **sample size** estimate even if no statistical methods were used.	Yes	Results, Figures, Figure legends
Were any steps taken to minimize the effects of subjective bias when allocating animals/samples to treatment (e.g. **randomization procedure**)? If yes, have they been described?	Yes	Methods
Include a statement about **blinding** even if no blinding was done.	Not Applicable	
Describe **inclusion/exclusion criteria** if samples or animals were excluded from the analysis. Were the criteria pre-established?<br><br>If sample or data points were omitted from analysis, report if this was due to attrition or intentional exclusion and provide justification.	Yes	Resuts, Figure
For every figure, are **statistical tests** justified as appropriate? Do the data meet the assumptions of the tests (e.g., normal distribution)? Describe any methods used to assess it. Is there an estimate of variation within each group of data? Is the variance similar between the groups that are being statistically compared?	Yes	Results, Methods

Sample definition and in-laboratory replication	Information included in the manuscript?	In which section is the information available? (Reagents and Tools Table, Materials and Methods, Figures, Data Availability Section)
In the figure legends: state number of times the experiment was **replicated** in laboratory.	Not Applicable	
In the figure legends: define whether data describe **technical or biological replicates**.	Not Applicable	

Ethics

Ethics	Information included in the manuscript?	In which section is the information available? (Reagents and Tools Table, Materials and Methods, Figures, Data Availability Section)
Studies involving **human participants**: State details of **authority granting ethics approval** (IRB or equivalent committee(s), provide reference number for approval.	Not Applicable	
Studies involving **human participants**: Include a statement confirming that **informed consent** was obtained from all subjects and that the experiments conformed to the principles set out in the WMA Declaration of Helsinki and the Department of Health and Human Services Belmont Report.	Not Applicable	
Studies involving **human participants:** For publication of **patient photos**, include a statement confirming that consent to publish was obtained.	Not Applicable	
Studies involving experimental **animals**: State details of **authority granting ethics approva**l (IRB or equivalent committee(s), provide reference number for approval. Include a statement of compliance with ethical regulations.	Not Applicable	
Studies involving **specimen and field samples:** State if relevant **permits** obtained, provide details of authority approving study; if none were required, explain why.	Not Applicable	

Dual Use Research of Concern (DURC)	Information included in the manuscript?	In which section is the information available? (Reagents and Tools Table, Materials and Methods, Figures, Data Availability Section)
Could your study fall under dual use research restrictions? Please check biosecurity documents and list of **select agents and toxins** (CDC): https://www.selectagents.gov/sat/list.htm	Not Applicable	
If you used a select agent, is the security level of the lab appropriate and reported in the manuscript?	Not Applicable	
If a study is subject to dual use research of concern regulations, is the name of the **authority granting approval and reference number** for the regulatory approval provided in the manuscript?	Not Applicable	

Reporting

The MDAR framework recommends adoption of discipline-specific guidelines, established and endorsed through community initiatives. Journals have their own policy about requiring specific guidelines and recommendations to complement MDAR.

Adherence to community standards	Information included in the manuscript?	In which section is the information available? (Reagents and Tools Table, Materials and Methods, Figures, Data Availability Section)
State if relevant guidelines or checklists (e.g., **ICMJE, MIBBI, ARRIVE, PRISMA**) have been followed or provided.	Not Applicable	
For **tumor marker prognostic studies**, we recommend that you follow the **REMARK** reporting guidelines (see link list at top right). See author guidelines, under 'Reporting Guidelines'. Please confirm you have followed these guidelines.	Yes	Introduction, Results, Methods, Discussion
For **phase II and III randomized controlled trials**, please refer to the **CONSORT** flow diagram (see link list at top right) and submit the CONSORT checklist (see link list at top right) with your submission. See author guidelines, under 'Reporting Guidelines'. Please confirm you have submitted this list.	Not Applicable	

Data Availability

Data availability	Information included in the manuscript?	In which section is the information available? (Reagents and Tools Table, Materials and Methods, Figures, Data Availability Section)
Have **primary datasets** been deposited according to the journal's guidelines (see 'Data Deposition' section) and the respective accession numbers provided in the Data Availability Section?	Yes	Data Availibity
Were **human clinical and genomic datasets** deposited in a public access-controlled repository in accordance to ethical obligations to the patients and to the applicable consent agreement?	Not Applicable	
Are **computational models** that are central and integral to a study available without restrictions in a machine-readable form? Were the relevant accession numbers or links provided?	Yes	Data Availibity
If publicly available data were reused, provide the respective **data citations in the reference list**.	Yes	References
